# Optical next generation reservoir computing

**DOI:** 10.1038/s41377-025-01927-6

**Published:** 2025-07-21

**Authors:** Hao Wang, Jianqi Hu, YoonSeok Baek, Kohei Tsuchiyama, Malo Joly, Qiang Liu, Sylvain Gigan

**Affiliations:** 1 Laboratoire Kastler Brossel, École Normale Supérieure—Paris Sciences et Lettres (PSL) Research University, Sorbonne Université, Centre National de la Recherche Scientifique (CNRS), UMR 8552, Collège de France, 24 rue Lhomond, 75005 Paris, France; 2 State Key Laboratory of Precision Space-time Information Sensing Technology, Department of Precision Instrument, Tsinghua University, Beijing, 100084 China; 3 Department of Electrical and Electronic Engineering, The University of Hong Kong, Hong Kong, China; 4 Department of Information Physics and Computing, Graduate School of Information Science and Technology, The University of Tokyo, 7-3-1 Hongo, Bunkyo-ku, Tokyo 113-8656 Japan

**Keywords:** Applied optics, Optical techniques

## Abstract

Artificial neural networks with internal dynamics exhibit remarkable capability in processing information. Reservoir computing (RC) is a canonical example that features rich computing expressivity and compatibility with physical implementations for enhanced efficiency. Recently, a new RC paradigm known as next generation reservoir computing (NGRC) further improves expressivity but compromises its physical openness, posing challenges for realizations in physical systems. Here we demonstrate optical NGRC with computations performed by light scattering through disordered media. In contrast to conventional optical RC implementations, we directly and solely drive our optical reservoir with time-delayed inputs. Much like digital NGRC that relies on polynomial features of delayed inputs, our optical reservoir also implicitly generates these polynomial features for desired functionalities. By leveraging the domain knowledge of the reservoir inputs, we show that the optical NGRC not only predicts the short-term dynamics of the low-dimensional Lorenz63 and large-scale Kuramoto-Sivashinsky chaotic time series, but also replicates their long-term ergodic properties. Optical NGRC shows superiority in shorter training length and fewer hyperparameters compared to conventional optical RC based on scattering media, while achieving better forecasting performance. Our optical NGRC framework may inspire the realization of NGRC in other physical RC systems, new applications beyond time-series processing, and the development of deep and parallel architectures broadly.

## Introduction

Dynamical systems, which receive external stimuli and responsively react to them, possess remarkable capacity to manipulate and process information^1,2^. As a nonlinear dynamical system, reservoir computing (RC) often builds upon recurrent neural networks (RNNs), utilizing the dynamics of internal reservoir states by a weighted summation to achieve desired functionalities^3,4^. The concept of RC not only finds various applications in time series forecasting^3,5,6^, classification^7^, prediction^8^, attractor manipulation^9^ and robots control^10^, but also connects computing theory, machine learning, neuroscience, biology and physics broadly^11^.

**Fig. 1:**
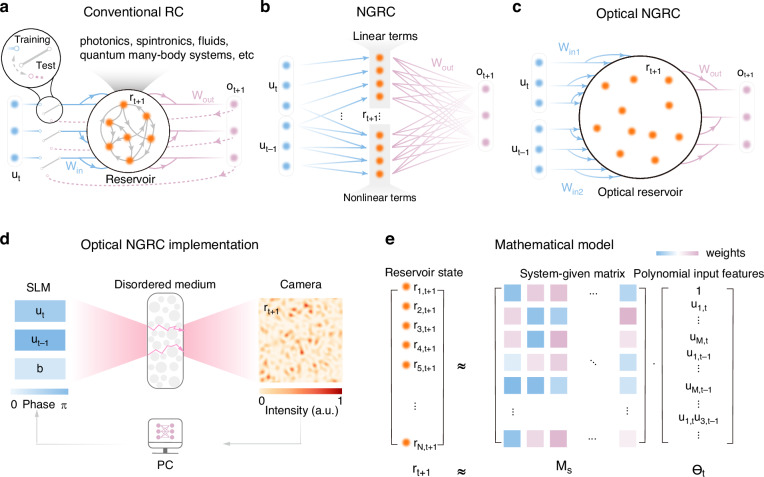
Optical next generation reservoir computing. **a** In the training phase, RC sequentially maps the current input (***u***_*t*_, blue) and the current reservoir state (***r***_*t*_) into the next reservoir state (***r***_*t*+1_, orange). After that, only a linear readout layer ***W***_*out*_ is trained to match ${\hat{{\boldsymbol{o}}}}_{t}={{\boldsymbol{W}}}_{out}{{\boldsymbol{r}}}_{t}$ with the desired output (***o***_*t*_, purple), which is often the input (i.e., ***o***_*t*_ = ***u***_*t*_) in the time series prediction tasks. In the prediction phase, by feeding back the predicted output to the input, RC can autonomously evolve as a dynamical system. **b** Different from the conventional RC scheme, next generation reservoir computing (NGRC) directly synthesizes reservoir features by constructing the polynomial functions of the time-delayed inputs (e.g., ***u***_*t*_ and ***u***_*t*−1_), without relying on an actual reservoir. **c** Similar to the digital NGRC, the proposed optical NGRC also drives the optical reservoir with time-delayed inputs. The optical process generates the polynomial features of the inputs implicitly. **d** The schematic experimental setup for the optical NGRC. First, input data at the current and the previous time steps (***u***_*t*_ and ***u***_*t*−1_) as well as a bias ***b***, are encoded onto the phase front of a laser beam via a spatial light modulator (SLM). Then, the modulated coherent light illuminates a disordered scattering medium, which provides rich mixing of the input and generates speckle patterns at the output. Finally, the reservoir features are obtained by measuring the intensity of the speckles in a camera. A computer (PC) is used to interface the SLM and the camera, as well as implementing readout layer. **e** The mathematical model of optical NGRC

What makes RC so appealing is, in part, its physical compatibility. A broad range of physical mechanisms and substrates have been harnessed to implement reservoirs (Fig. 1a)^12^, including analog electronics^13^, spintronic oscillators^14^, biological organoids^15^, and many more. All of these physical implementations aim at energy-efficient and high-throughput non-von Neumann architectures^16,17^. Among others, optical computing is of particular interest^18–20^, which employs photons as information carrier and light-matter interactions as processors, thereby exploiting the parallelism, energy efficiency and fast dynamics of light^21^. Within optical computing, optical RC has a history of exploration for over a decade^22–24^ and can be broadly classified into two types, i.e., delay-based reservoirs^23–31^ and spatial-distributed reservoirs^22,32–38^. The former relies on either a single^23–29^ or multiple^30^ nonlinear devices with time-delayed feedback to create virtual reservoir nodes in the time domain. The latter encompasses *in materia* reservoir systems built on semiconductor optical amplifiers^22^, integrated delay line networks^32^, diffractively coupled optical elements^33^, spatial light modulators (SLM) and cameras^34,35^, as well as multiple light scattering media^36–38^. Typically, these reservoirs can also be configured as a feedforward network commonly known as extreme learning machines^39^.

Often, new propositions on RC algorithms also influence and guide the designs of physical RC. For instance, a recent proposal of graph RC^40^ has been implemented in a topology of analog random resistive memory cells, achieving orders of magnitude higher energy efficiency compared to its digital courterpart^41^. Another example is the realization of deep RC networks in optics^42,43^, where the multi-timescale dynamics of stacked layers yields better computing performance^44^. Recently, a new RC paradigm, known as “next generation reservoir computing” (NGRC)^45^, has been proposed, which defines a reservoir feature directly from the domain knowledge of the original data^46^. True to its namesake, NGRC requires no more actual reservoirs for information mixing, but rather computes polynomial terms directly from the time-delayed inputs (Fig. 1b). Such digital NGRC has been trained to outperform traditional RC in benchmark forecasting and prediction tasks, even with less training data and time^45,46^. However, such a powerful architecture with growing prevalence in RC to date lacks physical realizations, partly due to the challenge of synthesizing these reservoir nodes explicitly. Implementing the NGRC algorithm in optical hardware may be advantageous by leveraging the exceptional scalability of optical reservoirs^37,47,48^.

In this work, we demonstrate an optical NGRC scheme based on light scattering through disordered media. Specifically, we drive our optical system with time-delayed inputs (Fig. 1c), as opposed to feeding current inputs and reservoir states in almost all previous physical RC implementations. Instead of generating polynomial features directly as in digital NGRC, such a refinement also allows the optical setup to produce expanded polynomial features, embedded in the generic high-dimensional speckle intensity representations (Fig. 1e). Optical NGRC features a multitude of advantages over conventional optical RC^37^ in processing time-series data. First, we demonstrate its efficacy in the short-term prediction of low-dimensional Lorenz63 and large-scale Kuramoto-Sivashinsky (KS) chaotic time series, achieving better prediction performance while using less than one tenth of the training data and a smaller reservoir compared to the previous state-of-the-art in optical RC^37^. Moreover, the optical NGRC also replicates the “climate” of the original manifolds in the long-term dynamics, which acts as a photonic surrogate model. Furthermore, we show that the optical NGRC can accurately infer unmeasured state variables in observer prediction applications, outperforming standard digital interpolation methods. The optical NGRC demonstrated in this work delivers more interpretable results by synthesizing features of time-delayed inputs through the optical system and then linearly combine them for versatile functionalities. Though our scheme is an indirect form of digital NGRC, it offers substantial compatibility with physical computing systems, thereby providing insights for tailoring various other physical reservoirs.

## Results

### Principle

We begin by briefly introducing the concept of RC, which is a RNN with fixed and random connectivity (Fig. 1a). For input data ${{\boldsymbol{u}}}_{t}=({u}_{1,t},{u}_{2,t},...,{u}_{M,t})\in {{\mathbb{R}}}^{M}$ and the internal reservoir states ${{\boldsymbol{r}}}_{t}=({r}_{1,t},{r}_{2,t},...,{r}_{N,t})\in {{\mathbb{R}}}^{N}$ at a given time *t*, the reservoir dynamics at the next time step evolves as:

1 $${{\boldsymbol{r}}}_{t+1}=f({{\boldsymbol{W}}}_{in}{{\boldsymbol{u}}}_{t}+{{\boldsymbol{W}}}_{r}{{\boldsymbol{r}}}_{t}+{\boldsymbol{b}})$$

where ***W***_*in*_ is the input matrix mapping input data to the neuron domain, ***W***_*r*_ is the interconnection matrix between neurons, ***b*** is the bias vector, and *f* is the activation function that is typically nonlinear. To further control the memory of RC, many architectures also incorporate an additional hyperparameter, know as the leaking rate, to balance the current nonlinear activation with the previous reservoir state. After evolving the reservoir for a sufficient time based on Eq. (1), a linear estimator can be trained to map the obtained reservoir states to the target outputs ***o***_*t*_ by defining ${\hat{{\boldsymbol{o}}}}_{t}={{\boldsymbol{W}}}_{out}{{\boldsymbol{r}}}_{t}\approx {{\boldsymbol{o}}}_{t}$, where ***W***_*out*_ is a readout layer mostly optimized through analytic linear regression (see Data processing in “Methods”). Note that in the time series prediction tasks, the desired output is often the input, i.e., ***o***_*t*_ = ***u***_*t*_. After training, the reservoir can autonomously evolve along a trajectory by closing the feedback loop in forecasting tasks. Importantly, the fixed nature of ***W***_*in*_ and ***W***_*r*_ renders RC a hardware-agnostic computing framework. RC also bypasses the challenges encountered in previous RNN training algorithms, such as backpropagation through time^1^, as it only trains the readout matrix ***W***_*out*_.

In contrast, the recently proposed NGRC builds the reservoir features directly from the input data in the polynomial form (Fig. 1b). While the polynomial order and the number of delayed inputs in NGRC are flexible and task-dependent, we formulate the NGRC with up to quadratic terms and inputs from two time steps for simplicity:

2 $${\boldsymbol{r}}_{t+1} = (1, \underbrace{\overbrace{u_{1,t},{\ldots},u_{M,t}}^{{\boldsymbol{u}}_t^T},\overbrace{u_{1,t-k},{\ldots},u_{M,t-k}}^{{\boldsymbol{u}}_{t-k}^T}}_{{\mathrm{Linear}}\,{\mathrm{terms}}}, \underbrace{\overbrace{u_{1,t}^2,{\ldots},u_{M,t}^2}^{{\mathbb{U}}({\boldsymbol{u}}_t \otimes {\boldsymbol{u}}_{t})},\overbrace{u_{1,t-k}^2,{\ldots},u_{M,t-k}^2}^{{\mathbb{U}}({\boldsymbol{u}}_{t-k} \otimes {\boldsymbol{u}}_{t-k})},\overbrace{u_{1,t}u_{1,t-k},{\ldots},u_{M,t}u_{M,t-k}}^{{\mathbb{U}}({\boldsymbol{u}}_t \otimes {\boldsymbol{u}}_{t-k})}}_{{\mathrm{Nonlinear}}\,{\mathrm{quadratic}}\,{\mathrm{terms}}})$$

where 1 denotes the bias term, ${{\boldsymbol{u}}}_{t-k}\in {{\mathbb{R}}}^{M}$ is a delayed input from *k* previous time steps (*k* = 1 is used hereafter unless otherwise specified). ⊗ denotes the outer product and ${\mathbb{U}}$ is defined as an operation to collect all unique monomials from the matrix vectorization of the outer product of two vectors.

With these in mind, we now adapt NGRC into a format that is compatible with optical implementations (Fig. 1c–e), such that we can use a similar optical setup as for conventional optical RC^36,37^ for NGRC. Our computing engine employs a continuous-wave laser as the light source, a phase-only SLM for data encoding, a linear scattering medium for information mixing, and a camera for feature detection (see setup in “Methods” and Supplementary Note 1). Here, the input data from different time steps is encoded onto the spatial phase profile of light via the SLM. The scattering medium linearly connects the input and output optical fields via a transmission matrix, mixing the input as speckle patterns at the camera plane. Then, the formation of speckle feature vectors is analogous to random projection, which is a ubiquitous computation tool widely used in mathematics and signal processing^19^. Taking into account the nonlinear responses of phase encoding of the SLM ($x\to \exp (ix)$) and square-law detection of the camera (*x* → ∣*x*∣^2^), the overall optical process defines the nonlinear mapping between the inputs (***u***_*t*_ and ***u***_*t*−1_) and the reservoir state (***r***_*t*+1_) as:

3 $${{\boldsymbol{r}}}_{t+1}=| {{\boldsymbol{W}}}_{in1}\exp (i{{\boldsymbol{u}}}_{t})+{{\boldsymbol{W}}}_{in2}\exp (i{{\boldsymbol{u}}}_{t-1})+{\boldsymbol{b}}{| }^{2}$$

where ***W***_*in*1_ and ***W***_*in*2_ are random complex matrices given by the optical scattering medium. In contrast to conventional optical RC schemes where the reservoir state at the time step *t* + 1 is calculated based on the current input ***u***_*t*_ and the reservoir state ***r***_*t*_^23–29,35–37^, we replace ***r***_*t*_ with the delayed input ***u***_*t*−1_ (Fig. 1d). Notably, this is different from the conventional RC framework augmented by delayed inputs^49^. Such a modification generates implicitly the polynomial forms of input variables at time steps *t* and *t* − 1 (Fig. 1e), as evident by expanding ***r***_*t*+1_ via Taylor series decomposition (see Supplementary Note 1):

4 $${\boldsymbol{r}}_{t+1} \approx {\boldsymbol{M}}_s \cdot [1, \underbrace{{\boldsymbol{u}}_t^T,{\boldsymbol{u}}_{t-1}^T}_{{\mathrm{Linear}}\,{\mathrm{terms}}}, \underbrace{{\mathbb{U}}({\boldsymbol{u}}_{t} \otimes {\boldsymbol{u}}_{t}), {\mathbb{U}}({\boldsymbol{u}}_{t-1} \otimes {\boldsymbol{u}}_{t-1}), {\mathbb{U}}({\boldsymbol{u}}_{t} \otimes {\boldsymbol{u}}_{t-1})}_{{\mathrm{Quadratic}}\,{\mathrm{terms}}}, {\ldots}]^T$$

where ***M***_*s*_ is a matrix given by the optical system, which mixes the underlying polynomial terms (**Θ**_*t*_) embedded in the speckle feature vector. In essence, due to the combined effects of phase encoding, mode mixing and intensity detection, the speckle vector can be understood as weighted sums of linear, quadratic and higher-order polynomial terms of ***u***_*t*_ and ***u***_*t*−1_. Stated differently, our optical system can compute similar feature terms just as the NGRC does in Eq. (2), only that an additional matrix linearly couples all these explicit terms together. Besides, the optimized linear readout matrix ***W***_*out*_ trained in optical NGRC can be related to ${{\boldsymbol{W}}}_{out}^{{\prime} }$ in digital NGRC by ${{\boldsymbol{W}}}_{out}^{{\prime} }\approx {{\boldsymbol{W}}}_{out}{{\boldsymbol{M}}}_{s}$, thus validating that our optical implementation is equivalent to the digital NGRC operation. Such an equivalence is further verified in the simulation, where we decompose the intensity of speckle nodes into sinusoidal functions (see Supplementary Note 1). This choice of basis arises from the nature of our optical system with phase encoding and intensity detection. Although it differs from the conventional polynomial function basis, it does not contradict the fundamental principle of NGRC^45^. Due to the presence of the system-given matrix ***M***_*s*_, the optical implementation operates in an indirect manner. Compared to conventional optical RC, this indirect way of implementing NGRC optically may also inherit the interpretability advantage of digital NGRC^45^. The reservoir computations can be understood through the synthesized features of the time-delayed inputs, which are then linearly combined by the readout layer ${{\boldsymbol{W}}}_{out}^{{\prime} }$ to perform specific tasks (see Supplementary Note 1).

### Forecasting Lorenz attractor

**Fig. 2:**
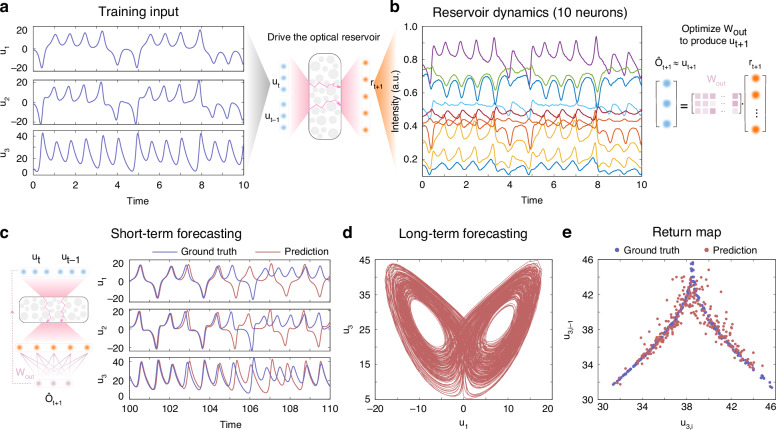
Optical NGRC for Lorenz63 attractor forecasting. **a** Time series of the Lorenz63 attractor (state variables *u*_1_, *u*_2_, *u*_3_) that drives the optical NGRC. At each time step of the training phase, the input states from the current (***u***_*t*_) and the previous (***u***_*t*−1_) time steps are encoded to the optical system to generate reservoir features (***r***_*t*+1_). **b** The temporal evolution of 10 randomly selected optical reservoir nodes (out of 2000 nodes), which resembles the dynamics of the input data. After training iterations of 4000 time steps, a linear estimator ***W***_*out*_ is trained to match the weighted sums of the reservoir features (${\hat{{\boldsymbol{o}}}}_{t}={{\boldsymbol{W}}}_{out}{{\boldsymbol{r}}}_{t}$) with the input data at the next time step (***u***_*t*_), i.e., ${\hat{{\boldsymbol{o}}}}_{t}\approx {{\boldsymbol{u}}}_{t}$. **c** Once ***W***_*out*_ is optimized, the optical NGRC is switched to the autonomous mode and experimentally predicts short-term results for 400 time steps. The normalized root mean square error (NRMSE) over the first 5 time units of the prediction phase is 0.0971. **d** The optical NGRC projects onto an attractor similar to the Lorenz63 attactor, experimentally obtained by the long-term forecasting results of 8000 time steps. **e** The return map of the ground truth (blue) and the experimental prediction (red)

To demonstrate the effectiveness of the proposed optical NGRC, we firstly apply our setup to the low-dimensional Lorenz63 time series forecasting task (see dataset information in “Methods”). As illustrated in Fig. 2, we initially drive the optical system by encoding ${[{{\boldsymbol{u}}}_{t},{{\boldsymbol{u}}}_{t-1}]}^{T}$ with a time interval of Δ*t* = 0.025 onto the SLM and we gather in total 4000 reservoir speckle feature vectors used for training (see experimental details in “Methods”). Figure 2b showcases the dynamics of 10 reservoir neurons measured in the experiment, providing nonlinear representations that reflect the characteristics of the input dataset. The smoothness of the reservoir dynamics, essential for reliable RC training, is guaranteed by the high stability of our experimental setup (see Supplementary Note 2). Then, we regress a digital readout layer ***W***_*out*_ to map the reservoir state ***r***_*t*_ to the next time step in the Lorenz63 attractor, i.e., ${\hat{{\boldsymbol{o}}}}_{t}={{\boldsymbol{W}}}_{out}{{\boldsymbol{r}}}_{t}\approx {{\boldsymbol{u}}}_{t}$ (see training details in “Methods”). After ***W***_*out*_ is obtained, the optical NGRC is used as an autonomous dynamical system for predicting another 400 time steps (see Supplementary Algorithm 1 and Supplementary Note 6 for task description). The training and prediction are implemented in the optical experiment with an effective system frame rate of around 10 Hz (see Supplementary Note 2).

In the short term, the optical NGRC shows decent forecasting capability of the Lorenz63 time series up to around 4 time units (Fig. 2c). Note that due to the nature of the chaotic systems, the prediction by optical NGRC would eventually diverge after a certain period of time, just as all models predicting chaos. Such a divergence does not imply the collapse of the RC model, rather, the ergodic (statistical) properties of the attractor are still preserved by RC, known as ‘climate’ replication^50,51^. To this end, we run the trained optical NGRC for an extended period of 8000 time steps. The long-term prediction consistently reproduces the manifold, as evident by the phase-space trajectory with double wings shown in Fig. 2d. Beyond visual inspection, we quantitatively evaluate the long-term forecasting performance by calculating the return map, in which the successive maxima of the third dimension ***u***_3_ in time are collected and plotted. As shown in Fig. 2e, the experimentally obtained data points collectively cluster around the ground truth curve, albeit with a deviation due to the presence of the experimental noise and quantization.

### Forecasting Kuramoto-Sivashinsky time series

**Fig. 3:**
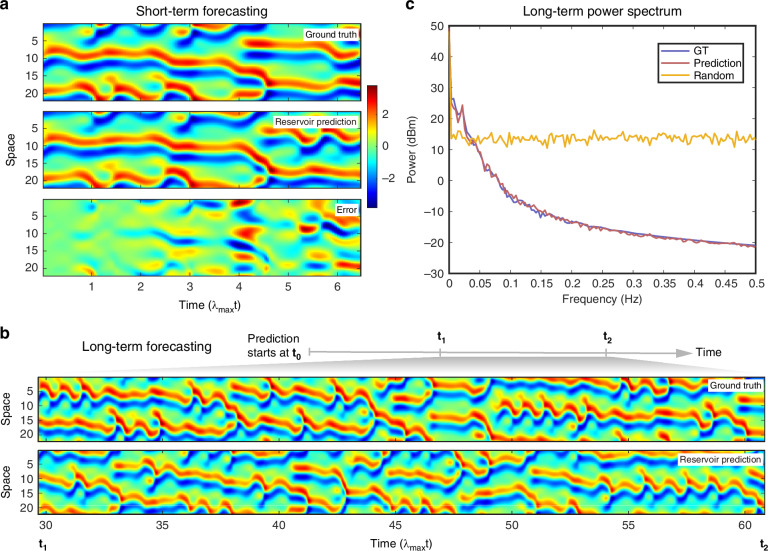
Optical NGRC for Kuramoto-Sivashinsky time series forecasting. **a** Experimental short-term prediction results of the Kuramoto-Sivashinsky (KS) time series with a domain size of *L* = 22 and a spatial sampling of *S* = 64. An optical NGRC with 2500 optical reservoir nodes is used for KS forecasting, which employs the current (***u***_*t*_) and the previous (***u***_*t*−1_) time steps in each training iteration for a total training length of 6000 time steps. The error subfigure (bottom) is the element-wise difference between the ground truth (top) and the experimental prediction (middle). The temporal axis is normalized by its largest Lyapunov time (*λ*_*max*_ = 0.043). **b** A part of the long-term prediction results by optical NGRC (between *t*_1_ and *t*_2_, where the prediction starts at *t*_0_). Albeit the complete deviation between the KS ground truth (top) and the optical NGRC predicted output (bottom) at the element-wise level, the optical NGRC replicates the long-term behavior of the KS chaotic system. **c** The power spectra of the long-term prediction in (**b**) (red), the KS ground truth (blue) and a random noise signal (yellow). The power spectra of the ground truth and optical NGRC predictions are in good agreement, in stark contrast to the power spectrum of the random noise background

Next, we use the optical NGRC in a more challenging scenario by forecasting another standard benchmark dataset in RC, i.e., a large-scale spatiotemporal chaotic KS time series (see dataset information in “Methods”). In Fig. 3a, we illustrate the short-term prediction results obtained in the experiment through online Bayesian optimization (see Data processing in “Methods” and Supplementary Note 2). After training the optical NGRC, the optical system can forecast the KS system reasonably well up to around 4 Lyapunov times (see definition in “Methods”), longer than the 2.5 Lyapunov times achieved previously^37^. At the prediction phase, the normalized root mean square (NRMSE) over the test period (6.45 Lyapunov times) is calculated as 0.2988 (see definition in “Methods”). We remark that, conventional RC typically necessitates a quite long warm-up period ranging from 100 to 100000 time steps^51^, which can be challenging in situations where training data is limited or the physical evolution of the reservoir system is time-consuming. Thanks to the NGRC operation^45^, we use in this study only 2 time steps for warm up and 6000 time steps for training, much shorter than the total 90500 time steps used in ref.^37^. To obtain more statistical prediction results, we conduct additional numerical simulations to validate the performance improvements of optical NGRC (see Supplementary Note 3, Supplementary Figs. S7–S9). Taken together, the better prediction performance in optical NGRC, combined with much less warm up and training data as well as a smaller reservoir size, collectively suggest the superiority of the optical NGRC over the conventional optical RC based on scattering media.

Regarding its long-term prediction performance, we illustrate in Fig. 3b a section (spanning from *t*_1_ to *t*_2_) of predicted outputs for 10000 time steps (starting at *t*_0_) beyond the short-term regime. While the prediction completely deviates from the ground truth at the element-wise level, the visual inspection indicates that the optical NGRC captures the correct “climate”^50^. We substantiate this observation by quantitatively analyzing the power spectra of the predicted outputs, the KS ground truth and a random noise signal in Fig. 3c (see data processing details in “Methods”). The long-term prediction results presented in Figs. 2 and 3 indicate that the optical NGRC effectively synchronizes with host prototypical systems, functioning as a physical twin without knowing their models.

### Optical NGRC observer

**Fig. 4:**
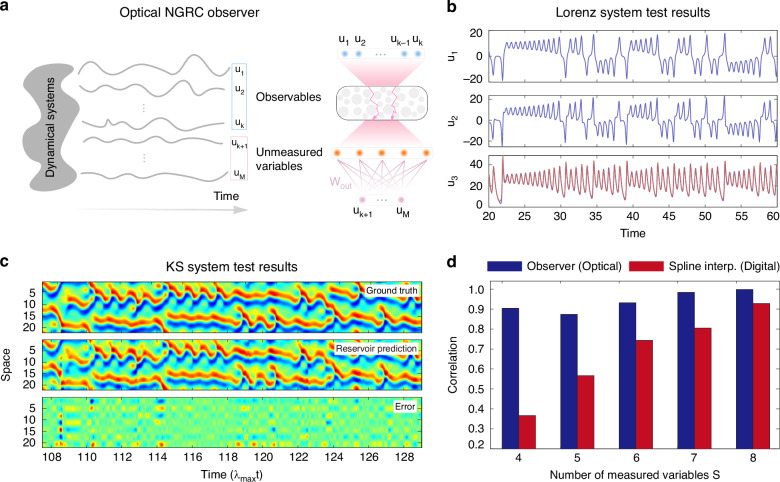
Optical NGRC observer. **a** For a dynamical system, often partial information of the full state of the system is measurable, e.g., state variables ${[{u}_{1},...,{u}_{k}]}^{T}$ are observables while ${[{u}_{k+1},...,{u}_{M}]}^{T}$ are unmeasured. The optical NGRC extracts information from measured observables (blue) and predicts unmeasured variables (purple) based on the state of the reservoir (orange). **b** Two variables *u*_1_ and *u*_2_ (blue) of the Lorenz63 system are provided as observables to infer the third variable *u*_3_. The predicted output by optical NGRC observer (red) matches the ground truth (blue) with high accuracy (NRMSE = 0.0169). **c** The optical NGRC observer results of the KS time series. 7 out of 64 spatial grids (evenly spaced in the spatial dimension) are input of the optical NGRC to infer the remaining 57 unmeasured variables. Top: ground truth; Middle: reservoir prediction (also including the observables for clarity); Bottom: error. **d** Performance comparison of the optical NGRC observer and the spline interpolation on the KS time series. The Pearson correlation between the optical NGRC observer prediction and the ground truth is consistently higher than that between the spline interpolation and the ground truth

We now proceed to the application of optical NGRC in a third benchmark task, referred to as the “reservoir observer”^8,45^. As illustrated in Fig. 4a, in many contexts when studying a dynamical system, it is common to have access to only a partial set of its complete degrees of freedom at a given time. An “observer” aims to deduce unmeasured variables from the measured ones (i.e., observables), for example, ${{[{u}_{1},...,{u}_{k}]}^{T}\,\,}^{\underrightarrow{{\rm{optical}}\,{\rm{NGRC}}}}\,\,{[{u}_{k+1},...,{u}_{M}]}^{T}$ (see Supplementary Algorithm 2). As before, we first train the optical reservoir in a supervised fashion, based on the limited number of time measurements where the full state variables of the system ${[{u}_{1},...,{u}_{k},...,{u}_{M}]}^{T}$ are accessible. Here, we conduct optical NGRC observer experiments on both the Lorenz63 and KS systems.

To follow the convention of the observer task in digital NGRC^45^, we employ one current and three delayed inputs to infer unmeasured variables, rather than the two inputs used in previous autonomous forecasting tasks. These four inputs are uniformly sampled with a stride of five time steps (see details in Supplementary Algorithm 2). For the Lorenz63 system, we infer *u*_3_ from *u*_1_ and *u*_2_. Figure 4b shows that only a short period of training time with 400 time steps yields decent predictions for 20 < *t* < 60, manifesting the feasibility of our optical NGRC in this application. Going beyond, we investigate the optical NGRC observer in the spatiotemporal KS system, based on the sparse spatial information that is available. Specifically, for the domain size of *L* = 22 of the KS time series studied in this work, we sample 64 spatial points at each time step. The experiments are performed using the knowledge of uniformly sampled *S* spatial state variables to infer the remaining set of 64—*S* variables. Figure 4c presents the test results when *S* = 7. We also vary *S* from 4 to 8 and summarize the calculated correlation between the experimental results and the corresponding ground truth in Fig. 4d. To provide a digital baseline, the cubic spline interpolation method is also implemented (see data processing and performance metrics details in “Methods”). We observe that the optical NGRC observer consistently outperforms the spline interpolation, serving as an effective means to reconstruct unmeasured dynamical system variables.

## Discussion

Generally, for physical RC systems, experimental factors such as device quantization and noise can limit the RC performance. Nevertheless, these requirements are not overly stringent for optical NGRC. In Supplementary Note 4, we show that 7- or 8-bit depth SLM and camera are sufficient for the tasks demonstrated in this work. To reduce the effect of the noise, we optimize the setup and average the experimental measurements when deriving the reservoir states (see Supplementary Note 2). The experimental results are comparable to numerical results reported in previous works (see Supplementary Table S3). Beyond forecasting chaotic time series, it is interesting to explore other machine learning tasks with optical NGRC, such as speech recognition^52^ and graph classification^40,44^. At the architecture level, the optical NGRC can also be chained to create more advanced neural networks like deep or parallel NGRC^44,53^ for enhanced expressivity and versatile functionalities. For instance, we can employ a thin scattering medium to only introduce the random mixing locally, so as to implement parallel optical NGRC^53^.

It is important to note that many steps in our current implementation involve processing in a digital computer like many other optical RC systems. Upgrading the system to an all-optical version remains a promising challenge, which could further improve the energy efficiency and computing throughput. Currently, we employ only a small fraction of pixels of the encoding SLM and detection camera. As a result, the speed advantage over digital counterpart is not shown in the current work. Nevertheless, our optical NGRC system can be scaled to accommodate larger inputs and larger reservoir output dimensions, allowing us to explore the potential scaling advantages of optical computing with scattering media as discussed in refs. ^37,54,55^.

Compared to digital NGRC, the scaling of optical NGRC and consequently the computational complexity, exhibits notable distinctions. Specifically, digital NGRC features polynomial scaling with respect to the input data dimension (see Supplementary Note 5), provided that dimension reduction of input data is not performed based on any prior knowledge^45,46^. This could impose a progressively heavier burden for digital NGRC to process large-scale systems. For example, for the same KS time series prediction studied in this work, the original digital NGRC scheme would require a reservoir size of 8385 when considering up to second-order polynomial terms, while we only use 2500 in optical NGRC (see Supplementary Note 5). Moreover, these 8385 polynomial functions may not produce prediction results comparable to those obtained by optical NGRC. More interestingly, due to the use of phase encoding and intensity detection in the optical NGRC, higher-order polynomials functions of delayed inputs (beyond the second-order due to Taylor expansion) are naturally embedded in every speckle grain mode, and they can be readily accessed by the linear readout layer if needed. For certain tasks that require predominantly low-order polynomials, the presence of higher-order polynomials in our system can be redundant. In such cases, we can increase the reservoir size to balance useful and unnecessary terms. Nevertheless, all the generated polynomials can form a valuable library that may be beneficial for general tasks without prior knowledge. As such, we do not have to manually determine the needed polynomial order or select the terms as necessitated in digital NGRC. In our case, we may use the phase encoding range as another hyperparameter to optimize the reservoir.

In summary, we propose and experimentally demonstrate in this work an efficient optical NGRC scheme based on light scattering through disordered media. Similar to the spirit of digital NGRC, optical NGRC generates the mixture of polynomial functions of time-delayed inputs. We leverage these features embedded in optical speckles for various benchmark tasks in RC, ranging from short-term and long-term forecasting to reservoir observer in Lorenz63 and KS chaotic systems. Optical NGRC features several advantages over conventional optical RC based on scattering media in significantly shorter training length, fewer hyperparameters, increased interpretability and greater expressivity, and may hold the prospect in scalability compared to digital NGRC towards large-scale chaotic systems (see Supplementary Table S1). Broadly, the proposed optical NGRC framework is hardware-agnostic, which could inspire new possibilities for a wide variety of physical RC substrates.

*Note Added*. During the finalization of the manuscript, we became aware that a related work on optical NGRC observer based on Rayleigh scattering was uploaded to arXiv^47^. A later work demonstrated photonic NGRC in an integrated photonics platform^48^. While refs. ^47,48^ focuses on the small-scale observer tasks, which is only the third part of our work, we deal with much more challenging short-term and long-term forecasting tasks involving both low-dimensional and large-scale chaotic systems (see Supplementary Note 6 for task comparisons).

## Materials and methods

### Experimental setup

The optical NGRC system (Supplementary Fig. 1) is primarily composed of a continuous-wave laser, an SLM, a disordered medium and a camera. The light from a low-power (2.5 mW) polarization-maintaining laser at 635 nm (Thorlabs, S1FC635PM) is delivered to a pinhole via a fiber. After the free-space propagation for a diffraction length of 100 mm from the pinhole, the input laser beam is collimated by a lens (L1, *f* = 100 mm). A polarizing beam splitter is used to match the output beam polarization with the working-axis of the following reflective phase-only SLM (Meadowlarks, HSP512L-1064). The input states are encoded onto the spatial wavefront of the laser beam. The modulated beam then passes through a 4 − *f* relay system (L2, *f* = 100 mm; L3, *f* = 100 mm) to reach the front surface of the scattering medium. In the experiment, we use a ground glass diffuser as the scattering medium, which is prepared by sandblasting the surface (*ϕ* 22 mm) of a microscope coverslip (1.5H, *ϕ* 25 mm, Deckgläser) with 220 grit white fused alumina. The full width at half maximum (FWHM) scattering angle of the diffuser is ~10 degrees. After the scattering process, the laser beam propagates freely for a length of 125 mm. The combined effects of multiple scattering and free-space propagation generate the reservoir states containing rich information of the inputs. The reservoir states are in the form of speckle patterns and are captured by a CMOS camera (Basler, acA1920-40um).

### Lorenz63 attractor, Kuramoto-Sivashinsky equation and Lyapunov exponents

The Lorenz63 attractor is a canonical chaotic manifold representing a simplified model of a weather system proposed by Lorenz in 1963, described by three ordinary differential equations:

5 $$\begin{array}{lll}\dot{{u}_{1}}\,=\,\sigma ({u}_{2}-{u}_{1})\\ \dot{{u}_{2}}\,=\,{u}_{1}(\rho -{u}_{3})-{u}_{2}\\ \dot{{u}_{3}}\,=\,{u}_{1}{u}_{2}-\beta {u}_{3}\end{array}$$

where *σ*, *ρ*, and *β* determine the system dynamics and ${[{u}_{1,t},{u}_{2,t},{u}_{3,t}]}^{T}$ is the system state variables at time *t*. In this work, we use the parameters *σ* = 10, *ρ* = 28 and *β* = 8/3, which gives rise to rich and chaotic dynamics that evolves on the double-wing attractor in the phase space. We integrate the equations using a fourth-fifth order Runge-Kutta method with a time step of Δ*t* = 0.025.

The KS equation is a partial differential equation that models many nonlinear systems with intrinsic instabilities, such as hydrodynamic turbulence and wave propagation in chemical reaction-diffusion systems. In this equation, dynamics at different scales interact mutually to generate spatiotemporal complexity governed by:

6 $${\partial }_{t}u+{\partial }_{x}^{4}u+{\partial }_{x}^{2}u+u{\partial }_{x}u=0$$

where the field *u*(*x*, *t*) is periodic on the spatial domain 0 ≤ *x* < *L*, that is *u*(*x*, *t*) = *u*(*x* + *L*, *t*) with *L* representing the spatial domain size. As the domain size *L* increases, the KS evolution changes rapidly. We use *L* = 22 in this study, which offers sufficient chaotic dynamics. We integrate the system based on a fourth order time-stepping method, on a spatial sampling grid of 64 (*S* = 64) and a time step of Δ*t* = 0.25.

The knowledge of Lyapunov exponents represents the most basic yet pervasive measure of a dynamical system. In simple terms, a (global) Lyapunov exponent is the average rate at which the system diverges from its initial point in the phase space along one degree of freedom. Therefore, high-dimensional systems contain multiple Lyapunov exponents, collectively forming a Lyapunov spectrum. To calculate the spectrum, we initialize multiple orthogonal vectors in different directions as perturbations and evaluate their average divergences along evolution compared to the dynamics without perturbations. In particular, the largest Lyapunov exponent *λ*_*max*_ serves as an effective indicator to evaluate whether the system exhibits chaotic behavior (*λ*_*max*_ > 0) or non-chaotic behavior (*λ*_*max*_ < 0). Multiplying time by *λ*_*max*_ yields the Lyapunov time in Figs. 3, 4, which denotes the average duration for errors to grow by a factor of *e*. For the Lorenz63 attractor, *λ*_*max*_ = 0.91. For the KS equation studied in this work, *λ*_*max*_ = 0.043.

### Data processing in the experiments

Here we provide more details of data encoding and processing used in this study. First, we normalize the Lorenz63 and KS time series to the range of [0, 1] with respect to their global minimum and maximum values. In the experiments, we linearly scale the normalized input data to [0, *π*] and encode it to the phase of light via an SLM. For short-term prediction, we apply two consecutive time steps, i.e., the current and previous inputs ***u***_*t*_ and ***u***_*t*−1_, to forecast the next step evolution (***u***_*t*+1_), and we introduce a relative weight *η* between these two inputs as a hyperparameter to potentially improve the performance. With another bias hyperparameter *b*, the input vector to be sent to the SLM is written as $\pi {[{{\boldsymbol{u}}}_{t},\eta {{\boldsymbol{u}}}_{t-1},b]}^{T}$. We remark that in this work we mostly use the phase range of [0, *π*] of the SLM, which results in an effective bit depth of 7 bits (0–127 in grayscale) for the encoding SLM. Practically, to reduce the crosstalk between pixels on the SLM, we use multiple pixels (macropixel) to represent each element of the input data vector above. Different macropixel sizes are used depending on the data dimensions (see Supplementary Table S2). In cases when the central region of the SLM is not entirely utilized, the unmodulated pixels serve as a static bias. To remove the unmodulated background light and unused periphery pixels from the SLM, we superimpose a diffraction grating mask over the encoded data mask. The grating is along the horizontal axis, consisting of alternating 0 and *π* phase levels with a 2 × 2 macropixel size. It diffracts the incoming beam into several diffraction orders and we select the first-order diffraction at the Fourier plane of the 4 − *f* system. We capture the speckle patterns within a predefined region of interest by the camera and downsample the measured images at intervals matching the speckle grain size, which is determined through speckle auto-correlation analysis. Subsequently, the speckle images are normalized from a range of 0–255 (8 bits) to a range of 0–1. We then randomly select independent nodes as needed from the normalized image, and flatten them into a reservoir feature vector for the following digital readout layer. Beyond random selection, more advanced feature selection methods can be employed to further enhance the prediction performance and reduce the readout complexity. To improve the forecasting performance, we concatenate the reservoir state and the current input state for prediction as applied in previous works^37^. Note that this concatenation process is not strictly needed for optical NGRC implementation in this work, though it may help produce more stable experimental results. Afterwards, the predicted output is scaled back to the original data range based on the previously determined minimum and maximum values. We summarize all the aforementioned parameters used in the experiments in Supplementary Table S2.

Once sufficient training reservoir states are collected, we train a digital linear readout layer ***W***_*out*_ by the Tikhonov regularization method to map the reservoir states ***R*** to the targets ***O***. In particular, the optimal ***W***_*out*_ is computed through minimizing the following objective function:

7 $${{\boldsymbol{W}}}_{out}={\rm{argmin}}({\parallel {{\boldsymbol{W}}}_{out}{\boldsymbol{R}}-{\boldsymbol{O}}\parallel }_{2}^{2}+\beta {\parallel {{\boldsymbol{W}}}_{out}\parallel }_{2}^{2})$$

where *β* is the ridge regularization parameter to punish large weight values in ***W***_*out*_. The ridge regression can be computed efficiently via the explicit solution ${{\boldsymbol{W}}}_{out}={\boldsymbol{O}}{{\boldsymbol{R}}}^{T}{({\boldsymbol{R}}{{\boldsymbol{R}}}^{T}+\beta {\boldsymbol{I}})}^{-1}$, without the need of error backpropagation. *β* is an important hyperparameter which can improve the generalization ability and avoid overfitting, especially when the number of reservoir nodes is larger than the training length. When searching for the optimal *β* in the reservoir observer task, singular value decomposition of ***R*** can be used to further accelerate the computations.

For the quantitative analysis of the long-term power spectrum reported in Fig. 3c, we apply a sliding window approach similar to the short-time Fourier transform. Specifically, we sample one spatial grid point (32nd is used) from the 64 spatial grids of the spatiotemporal data to create a one-dimensional time series. Then we partition the time series of 10000 data points into 20 intervals, each comprising 500 data points. Subsequently, we calculate the corresponding power spectrum by Fourier transform for each interval, and average them over all intervals. In this way, we obtain smoother power spectra of the time series and avoid local oscillations. As for the random noise signal, we initialize it by drawing random numbers from a uniform distribution. In the figure, we only illustrate the positive frequency part of the power spectra since it is symmetric with the negative part.

To establish the digital baseline for the optical reservoir observer, we use the cubic spline interpolation method, which resorts to low-order polynomials for smooth and accurate fitting while mitigating high-order polynomial oscillations. To do so, we employ the *CubicSpline* function from the SciPy Python library with a periodic boundary condition.

For short-term forecasting of chaotic time series, we use the online Bayesian optimization approach, i.e., we run the optical NGRC setup on-the-fly during the hyperparameter optimization. This is an effective approach to achieve stable and reliable predictions from reservoirs with noise. Compared to other hyperparameter optimization techniques such as grid search or random search, Bayesian optimization is recently founded to be the optimal approach due to its fast convergence and effectiveness, especially in scenarios with large search spaces. Essentially, a probabilistic surrogate model is optimized to predict the optimal parameters based on observed data under given metrics. This probabilistic model explores new parameter spaces iteratively, which can effectively reduce the risk of getting trapped in local minima by considering both the predicted performance and the uncertainty of the model. In practice, we typically run 20–30 iterations using the *bayesOpt* library in MATLAB during experiments (see Supplementary Note 2).

For all experimental data collection, we use MATLAB software on a desktop equipped with an Intel(R) Core(TM) i7-6700 CPU and 32 GB RAM. For the data analysis and simulations, we use another desktop with an AMD EPYC 7351P CPU and 64 GB RAM.

### Performance evaluation metrics

Here we describe two metrics used in the data analysis and performance evaluation. The metric NRMSE used in this work is defined as ${\rm{NRMSE}}=\frac{1}{{O}_{\max }}\sqrt{\frac{\mathop{\sum }\nolimits_{i = 1}^{K}\mathop{\sum }\nolimits_{j = 1}^{P}{({\hat{o}}_{j,i}-{o}_{j,i})}^{2}}{KP}}$, where ${O}_{\max }$ represents the maximum value of the ground truth dataset *O*, *K* is the total number of time steps and *P* is the output size. This metric is useful to understand the overall performance across a certain period of the time series. When comparing the performance of optical reservoir observer with spline interpolation on the KS system, we use the Pearson correlation coefficient calculated as $r=\frac{\mathop{\sum }\nolimits_{i = 1}^{K}\mathop{\sum }\nolimits_{j = 1}^{P}({\hat{o}}_{j,i}-{\hat{O}}_{{\rm{mean}}})({o}_{j,i}-{O}_{{\rm{mean}}})}{\sqrt{\left[\mathop{\sum }\nolimits_{i = 1}^{K}\mathop{\sum }\nolimits_{j = 1}^{P}{({\hat{o}}_{j,i}-{\hat{O}}_{{\rm{mean}}})}^{2}\right]\left[\mathop{\sum }\nolimits_{i = 1}^{K}\mathop{\sum }\nolimits_{j = 1}^{P}{({o}_{j,i}-{O}_{{\rm{mean}}})}^{2}\right]}}$, with ${\hat{O}}_{{\rm{mean}}}({O}_{{\rm{mean}}})$ denoting the mean value of the predicted outputs (ground truth).

## Supplementary information

Supplementary Information for Optical next generation reservoir computing

## Data Availability

The data and codes that support the plots and reservoir computing simulations within the paper are available at https://github.com/comediaLKB/Optical-NGRC-based-on-multiple-light-scattering.
