# Supplementary material for: Optical next generation reservoir computing
Source: Light Sci Appl. 2025 Jul 21;14:245. doi: 10.1038/s41377-025-01927-6 (PMC12280216; doi:10.1038/s41377-025-01927-6)
Supplement: Supplementary file 1 — Supplementary Information for Optical next generation reservoir computing [file 41377_2025_1927_MOESM1_ESM.pdf]

Supplementary information for:
Optical next generation reservoir computing

Hao Wang[1,2,*], Jianqi Hu[1,4,*,†], YoonSeok Baek[1], Kohei Tsuchiyama[1,3], Malo Joly[1], Qiang Liu[2,†], & Sylvain Gigan[1,†]

[1]*Laboratoire Kastler Brossel, École Normale Supérieure - Paris Sciences et Lettres (PSL) Research University, Sorbonne Université, Centre National de la Recherche Scientifique (CNRS), UMR 8552, Collège de France, 24 rue Lhomond, 75005 Paris, France.*

[2]*State Key Laboratory of Precision Space-time Information Sensing Technology, Department of Precision Instrument, Tsinghua University, Beijing 100084, China.*

[3]*Department of Information Physics and Computing, Graduate School of Information Science and Technology, The University of Tokyo, 7-3-1 Hongo, Bunkyo-ku, Tokyo 113-8656, Japan.*

[4]*Department of Electrical and Electronic Engineering, The University of Hong Kong, Hong Kong, China.*

[*] *These authors contributed equally to the work*

[†] *Email: jianqi@hku.hk*

[†] *Email: qiangliu@tsinghua.edu.cn*

[†] *Email: sylvain.gigan@lkb.ens.fr*

Supplementary Notes:

1. The principle of optical NGRC
2. Experimental setup details
3. Simulation comparison of optical NGRC and optical conventional RC based on scattering media
4. The impact of device quantization and noise on optical NGRC
5. Optical computation analysis
6. Comparisons between one-step prediction and autonomous prediction

Supplementary Figures:

Figure S1. Optical NGRC experimental system
Figure S2. Optical NGRC principle
Figure S3. Illustration of optical NGRC formulation in matrix representation.
Figure S4. Comparison of the effective readout matrices of optical NGRC and digital NGRC in the same feature basis.
Figure S5. Experimental system stability
Figure S6. Bayesian optimization log during the short-term prediction of KS time series experiments
Figure S7. Simulated performance comparison of optical NGRC and optical conventional RC at different reservoir sizes
Figure S8. Simulation of optical NGRC forecasting errors based on different quantization bit depths of devices
Figure S9. Simulated performance comparison of optical NGRC and optical conventional RC at different noise levels added to reservoir features
Figure S10. Illustration of differences between one-step prediction and autonomous prediction.

Supplementary Algorithms:

Algorithm S1. Optical NGRC for forecasting dynamical systems
Algorithm S2. Optical NGRC for deducing unmeasured variables of dynamical systems

Supplementary Tables:

Table S1. Comparison of optical NGRC, optical conventional RC based on scattering media and digital NGRC
Table S2. Summary of data encoding and processing parameters used in the experiments
Table S3. Performance comparison with previous works on Lorenz63 and KS time-series prediction

Supplementary Note 1. The principle of optical NGRC

Multiple scattering phenomenon in optics has recently been harnessed as a computational resource owing to its inherent complexity and high dimensionality[1]. As coherent laser light travels through a disordered medium, it experiences random scattering and numerous interference events occur. This process results in the formation of a speckle field at the output. For a given fixed scattering medium, the input and output optical fields are deterministically related by a complex matrix $\boldsymbol{W}$, known as the transmission matrix (TM), i.e., $\boldsymbol{E}_{out} = \boldsymbol{W} \cdot \boldsymbol{E}_{in}$. Therefore, despite the apparent randomness of the speckle field, it encapsulates rich information of the input as speckle features. Experiments and theoretical studies reveal that the real and imaginary parts of the entries in the TM follow Gaussian independent and identical distributions (see Supplementary Figs. S1b-c). This spurs the recent research interests in harnessing such a disordered optical process for signal processing tasks[1]. In essence, this process can be conceptualized as a matrix-vector multiplication, where the input data $\boldsymbol{E}_{in}$ is multiplied by a random matrix $\boldsymbol{W}$. Au such, it performs optically the random projection, a ubiquitous mathematical operation used in many signal processing scenarios[2]. The optical setup with multiple light scattering executes random projection in a fast way without the need to measure or digitally store the random matrix, which can reach an extreme scale where the benefits of optical computing become pronounced. Following this spirit, optical random projection has been successfully applied in diverse computing and signal processing tasks including reservoir computing[3,4], extreme learning[5], spin-glass simulator[6,7], reconfigurable linear operators[8], direct feedback alignment training[9], graph kernel[10], online change-point detection[11], among others.

In our optical system, we employ a phase-only SLM to encode input data $\boldsymbol{x} = [x_1, x_2, ..., x_M]^T$, a scattering medium to perform random mixing (feature extraction), and a camera to retrieve the resulting speckle intensity features (reservoir features) $\boldsymbol{y} = [y_1, y_2, ..., y_N]^T$. Although light scattering itself is fundamentally linear in this work, our system achieves a nonlinear mapping between the input data and the output (reservoir) features because of the strategic incorporation of phase encoding and intensity detection nonlinearities, as described by $\boldsymbol{y} = |\boldsymbol{W} \cdot \exp(i\boldsymbol{x})|^2$. Therefore, the combination of linear random projection and element-wise nonlinearity provides rich high or-

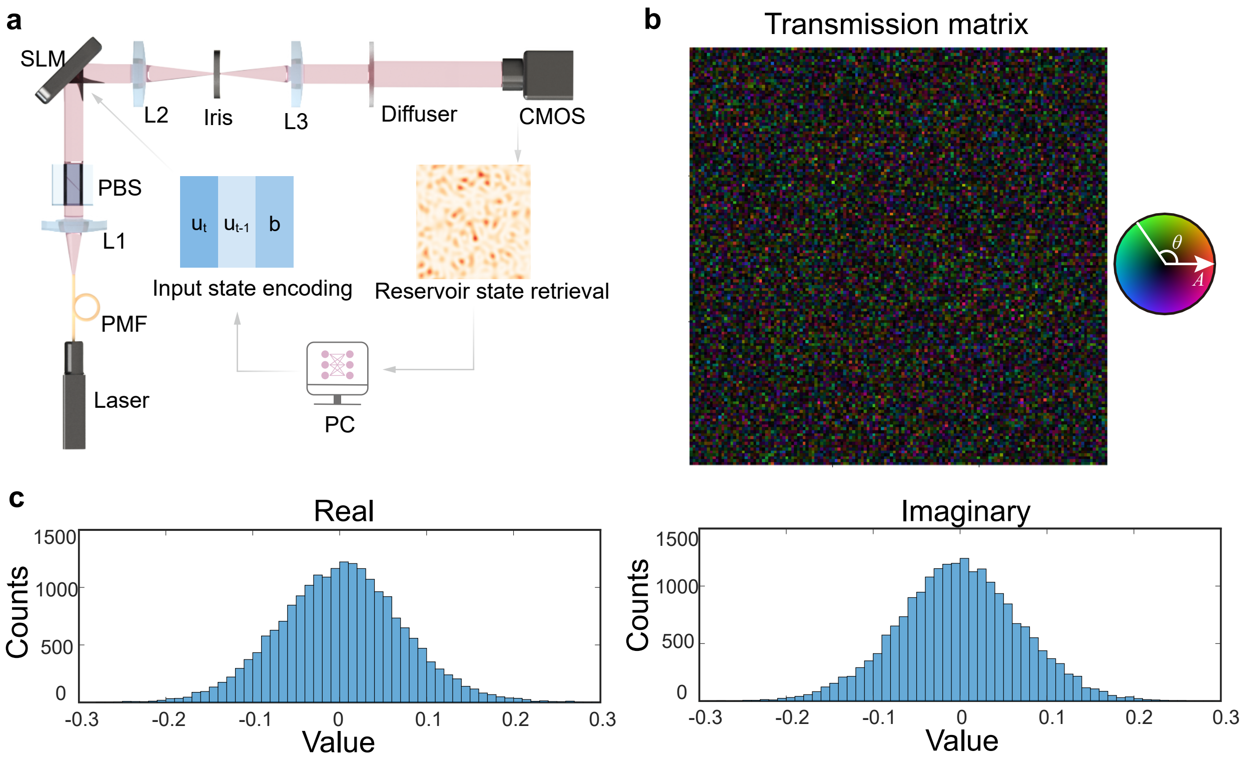

Figure S1: Optical NGRC experimental system. **a** Experimental setup. PMF: polarization-maintaining fiber; PBS: polarizing beam splitter; SLM: spatial light modulator; Diffuser: ground glass diffuser; CMOS: camera; L1, L2, L3: lens; PC: personal computer. **b** Typical experimental transmission matrix of the current setup (144×144). **c** Statistical distribution of the entries of the TM in **b** (left panel: real components; right panel: imaginary components).

der nonlinear terms, which can be used for the construction of NGRC. Collectively, we can rewrite the output intensity at the n-th output mode using Taylor expansion:

$$y^{(n)} \approx \alpha_{00}^{(n)} + \underbrace{\alpha_{10}^{(n)} x_1 + \alpha_{20}^{(n)} x_2 + \ldots}_{\text{Linear terms}} + \underbrace{\alpha_{11}^{(n)} x_1^2 + \ldots + \alpha_{12}^{(n)} x_1 x_2 + \ldots}_{\text{Quadratic terms}} + \ldots \tag{1}$$

where $\alpha_{ij}^{(n)}$ represents the weighted coefficient (here the nomenclature of the subscript is shown up to quadratic terms for clarity). The equation above implies that the optical nonlinear mapping of our setup is equivalent to firstly calculating the rich monomials (polynomial terms) of the input data explicitly, and then linearly combining them into speckle intensity features. This understanding is crucial to link optical NGRC in this work and digital NGRC[12]. Experimentally, by encoding

multiple time steps of input time series data into $\boldsymbol{x}$ (e.g., $[\boldsymbol{u}_t, \boldsymbol{u}_{t-1}]^T \rightarrow \boldsymbol{x}$), and specifying the reservoir state $\boldsymbol{r}_{t+1}$ at the time step $t+1$ from $\boldsymbol{y}$ in Eq. (1) ($\boldsymbol{y} \rightarrow \boldsymbol{r}_{t+1}$) as well as grouping all the coefficients $\alpha_{ij}^{(n)}$ into $\boldsymbol{M}_s$, we derive Eq. (4) in the main text. The explicit polynomial feature terms of the input data are compiled into a feature vector denoted by $\boldsymbol{\Theta_t}$. In other words, $\boldsymbol{\Theta_t}$ is the reservoir feature terms at the time step t of NGRC, namely $\boldsymbol{\Theta_t} = (1, \boldsymbol{u}_t^T, \boldsymbol{u}_{t-1}^T, \mathbb{U}(\boldsymbol{u}_t \otimes \boldsymbol{u}_t), \mathbb{U}(\boldsymbol{u}_{t-1} \otimes \boldsymbol{u}_{t-1}), \mathbb{U}(\boldsymbol{u}_t \otimes \boldsymbol{u}_{t-1}), \ldots)$ with $\mathbb{U}$ as an operation to collect all unique monomials from the matrix vectorization of the outer product of two vectors. And the system-given matrix $\boldsymbol{M}_s$ incorporates the phase encoding, TM, and intensity detection of the optical setup altogether. Upon formulating the reservoir state as $\boldsymbol{r}_{t+1} \approx \boldsymbol{M}_s \cdot \boldsymbol{\Theta_t}$, we optimize a linear digital readout layer $\boldsymbol{W}_{out}$ atop the reservoir states for prediction, i.e., $\boldsymbol{o}_t = \boldsymbol{W}_{out}\boldsymbol{r}_t \approx \boldsymbol{W}_{out}\boldsymbol{M}_s\boldsymbol{\Theta_t}$. This is equivalent to directly harnessing the polynomial feature terms $\boldsymbol{\Theta_t}$ by $\boldsymbol{W}'_{out}\boldsymbol{M}_s$, which is at the heart of NGRC. Note that when applying our system to forcasting time series such as Lorenz63 that only needs up to quadratic polynomial terms, higher-order polynomials become unnecessary and can be penalized by the trained readout matrix $\boldsymbol{W}_{out}$. Since the system-given matrix $\boldsymbol{M}_s$ is a fixed matrix for a certain system, training $\boldsymbol{W}_{out}$ is effectively training $\boldsymbol{W}'_{out}$, therefore augmenting the weights of useful terms and punishing unnecessary terms. We visualize the working principle of optical NGRC in Supplementary Fig. S2.

To illustrate how one can rewrite the optical random projection as Eq. (3) in the main text, we refer to Supplementary Fig. S3. As shown in this figure, an output mode is the dot product of a row of the transmission matrix and the input field vector. As such, we can break out the dot product into sub groups containing separately the current input, the previous input and the bias. Similarly, in the matrix form, the computation can also be divided into sub blocks, such that the input matrices $\boldsymbol{W}_{in1}$ and $\boldsymbol{W}_{in2}$ are related to $\boldsymbol{u}_t$ and $\boldsymbol{u}_{t-1}$ separately.

Our optical NGRC scheme can immediately inspire a wide array of physical NGRC based on other various physical substrates, especially for *in materia* RC[13]. Given a physical reservoir system that performs a nonlinear transformation on its input, say $\boldsymbol{y} = f(\boldsymbol{x})$, we can stimulate the system with time-delayed inputs using our recipe illustrated above, and record the output as reservoir

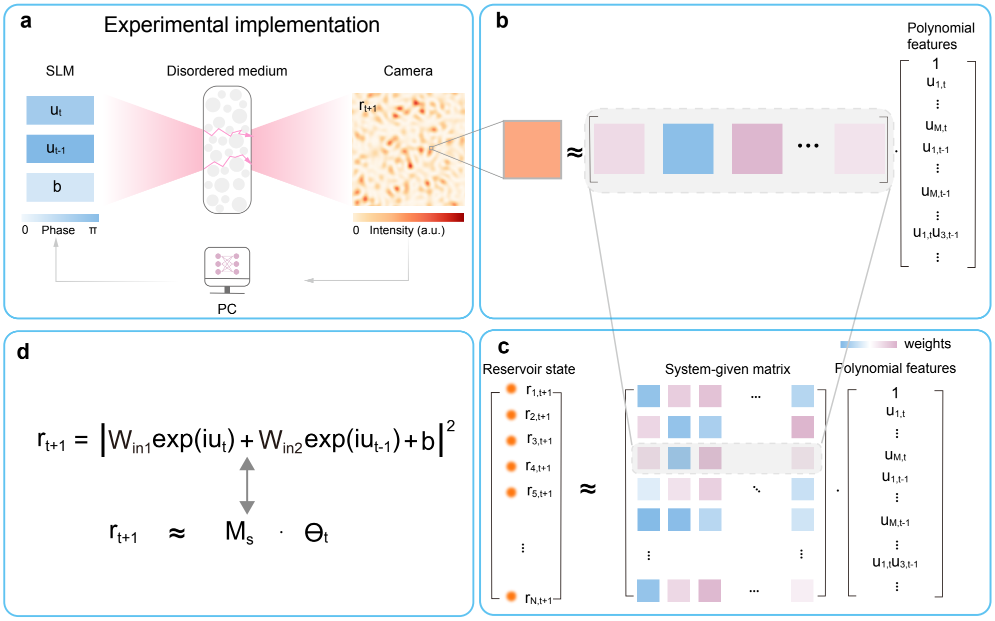

Figure S2: Optical NGRC principle. **a** Optical implementation. **b** Decomposition of the speckle intensity feature at an output mode. **c** Mathematical model of optical NGRC. **d** Matrix representation of optical NGRC.

features to define a physical NGRC. More specifically, the physical NGRC can be formulated as $\boldsymbol{r}_{t+1} = f(\boldsymbol{u}_t, \boldsymbol{u}_{t-1})$ (here only two delayed inputs are shown as an example). Through a similar decomposition analysis shown above, one can achieve:

$$\boldsymbol{r}_{t+1} \approx \boldsymbol{M}_s \cdot [1, \underbrace{\boldsymbol{u}_t^T, \boldsymbol{u}_{t-1}^T}_{\text{Linear terms}}, \underbrace{\mathbb{U}(\boldsymbol{u}_t \otimes \boldsymbol{u}_t), \mathbb{U}(\boldsymbol{u}_{t-1} \otimes \boldsymbol{u}_{t-1}), \mathbb{U}(\boldsymbol{u}_t \otimes \boldsymbol{u}_{t-1})}_{\text{Quadratic terms}}, ...]^T \tag{2}$$

where $\boldsymbol{M}_s$ is specified by the physical system, and the remaining symbols are consistent with the notation defined in the main text, e.g., $\mathbb{U}$ denotes an operation to collect all unique monomials from the matrix vectorization of the outer product of two vectors. Generalizing our optical NGRC model to a broader regime of physical NGRCs can open up many intriguing directions and hold the potential to enhance the performance of conventional physical RC across diverse scenarios. We emphasize that understanding the physical model $\boldsymbol{y} = f(\boldsymbol{x})$ is crucial before implementing the physical NGRC, as this mapping determines what terms are generated when injecting inputs like $\boldsymbol{r}_{t+1} = f(\boldsymbol{u}_t, \boldsymbol{u}_{t-1})$.

At the last part of this note, we quantitatively show that why our optical system is equivalent

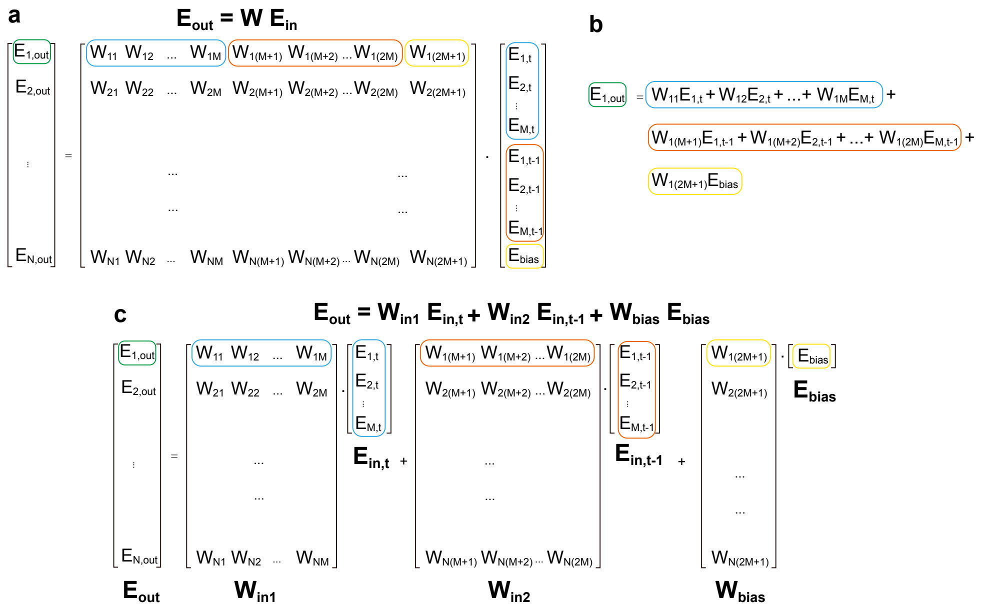

Figure S3: Illustration of optical NGRC formulation in matrix representation. a In our optical system, the input field and output field are linearly connected by a transmission matrix. **b** Every output element (the first is shown as an example) can be represented as the dot product of a row of the transmission matrix and the input field vector. The input elements include the current and previous states as well as the bias. **c** Similar to breaking out the dot product into sub groups, we can rewrite the matrix vector multiplication the way as presented in the main text.

to NGRC implementation. To this end,we perform additional simulation to investigate the underlying feature extraction process of the optical setup. We use the Lorenz63 autonomous forecasting as an example. The goal is to compare the weights of the readout matrix obtained in the optical approach with that of the digital NGRC computed in the same feature basis. For this specific task, we encode 7 macropixels to the SLM, including 3 for the current input ($u_{1,t}$, $u_{2,t}$, $u_{3,t}$), 3 for the previous input ($\eta u_{1,t-1}$, $\eta u_{2,t-1}$, $\eta u_{3,t-1}$) and a bias b. In the simulation, we use $\eta = 3$ and $b = 0$ as an example and the inputs are normalized to $[0, \pi]$, without loss of generality. We also consider the phase encoding ($x \rightarrow \exp(ix)$) of the SLM and intensity detection ($x \rightarrow |x|^2$) of the system. While the reservoir size may be large, the generated features of such an optical system can be described by the weighted sums of a number of sinusoidal functions. Here we decompose the speckle features into the sinusoidal function basis instead of the polynomial function basis, as these sinusoidal functions naturally arise from our optical system with phase encoding and intensity detection. This

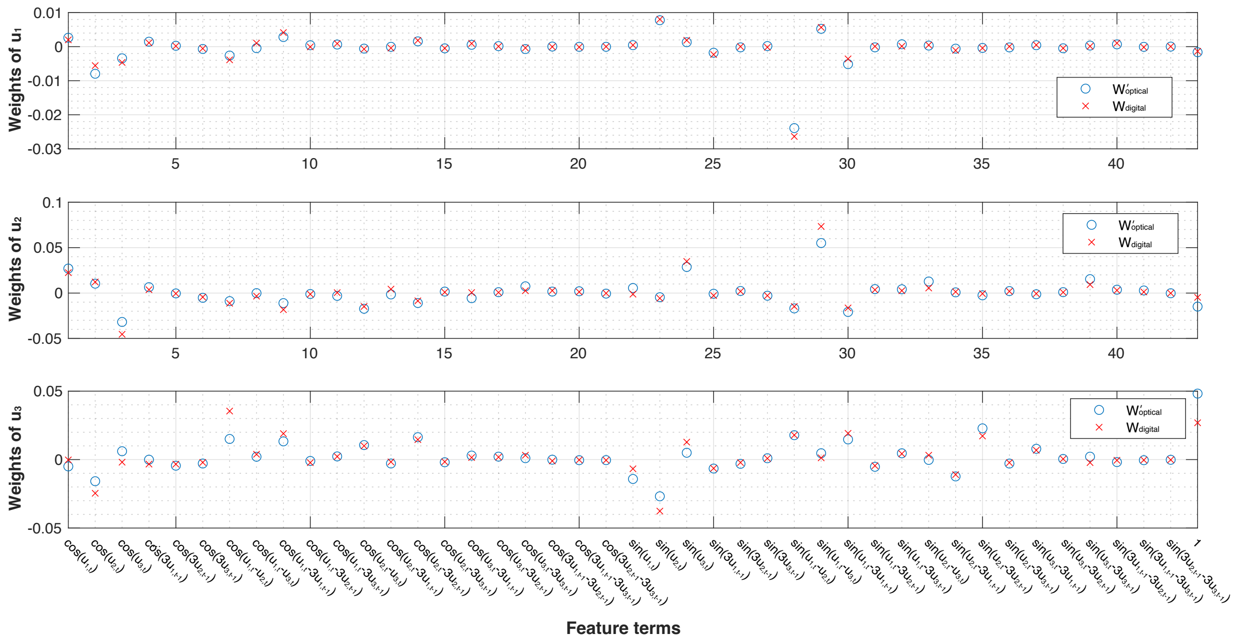

Figure S4: Comparison of the effective readout matrices of optical NGRC and digital NGRC in the same feature basis.

choice of the basis is justified by the insights from the original digital NGRC work, which indicates that the core idea of the algorithm is to leverage linear and nonlinear functions of delayed input data, without exclusively relying on the polynomial functions[12]. There are in total 43 independent feature terms, which are shown in the axis of Supplementary Fig. S4. These include 21 cosine terms and 21 sine terms as well as a bias term. To understand the origin of these 43 terms, one can recall the interference of two phasors with modular square operation, which yields DC terms and a cosine term with its argument being their phase difference. This process can be generalized to more modes and one can easily derive that there are in total 43 terms, by considering two steps of inputs (6 state variables) and a bias (note the bias here is also a phasor). For optical NGRC, we first compute the readout matrix $\boldsymbol{W}_{optical}$ and then project them in these 43 feature terms to obtain a new readout matrix $\boldsymbol{W}'_{optical}$. In this way, we can fairly compare the optical NGRC with digital NGRC that is built upon these feature terms directly, since they use the same feature basis. We then train a linear readout layer $\boldsymbol{W}_{digital}$ using digital NGRC. As clearly shown in Supplementary Fig.

S4, the close matching between $\boldsymbol{W}'_{optical}$ and $\boldsymbol{W}_{digital}$ confirms that the optical NGRC operates in the same way as digital NGRC.

Supplementary Note 2. Experimental setup details

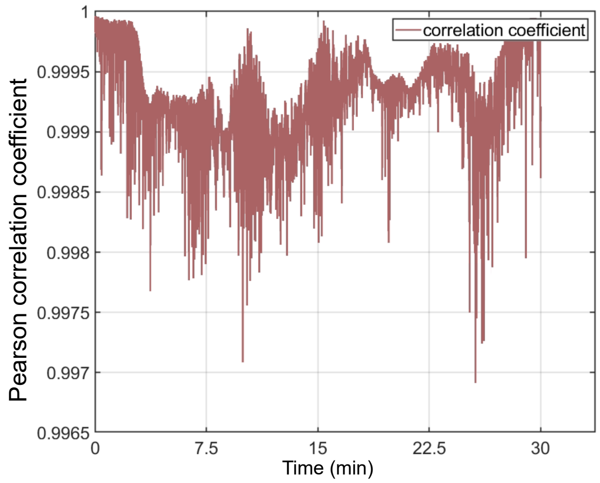

Figure S5: Experimental system stability. We calculate the correlations between the initial speckle intensity pattern frame and subsequent frames (reservoir features), all generated using the same phase mask in the SLM.

Here we describe additional experimental details to complement the Materials and Methods. The schematic diagram of the experimental setup is shown in Supplementary Fig. S1a and a representative TM is shown in Supplementary Fig. S1b. To ensure the accurate phase encoding of input data into optical signals, we calibrate the SLM by updating its lookup table at the optical wavelength of 635 nm. The stability of the optical setup is crucial for the predictions of chaotic time series in optical NGRC. To reduce the experimental noise, we optimize the setup in several aspects. Firstly, we utilize an air conditioner in the lab and a shielding cage enclosing the whole setup to ensure a stable ambient environment. We use only a small central region of the full screen of the SLM to reduce the influence of mechanical vibrations associated with the SLM. Additionally, we place another shielding cage covering both the SLM driver and flex cable to prevent turbulent air flows. A long tube is mounted in front of the camera to block ambient light. To further reduce the influence of noise, for a given phase mask, we repeat the acquisition 4 times

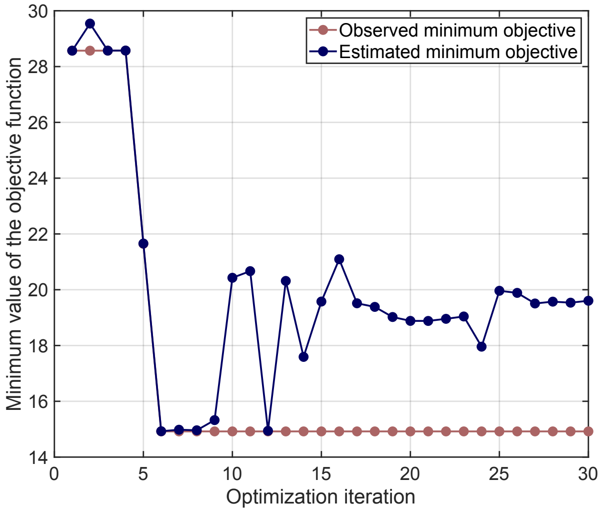

Figure S6: Bayesian optimization log during the short-term prediction of KS time series experiments. The objective function is defined as the accumulated error over a certain period of time in the prediction test. The red curve corresponds to the experimentally realized minimum objective function value (up to the number of iterations), while the blue curve indicates what the Bayesian model predicts or estimates, based on the previous experimental realizations.

and average the results to derive the actual reservoir state. The whole optical system operates at a frame rate of 40 Hz, therefore we can collect 10 reservoir state vectors per second. Taken together, the system is stable enough for consistent computations as an optical reservoir. To quantitatively evaluate the noise level, we collect multiple speckle images with the same input, and then calculate the ratio between the standard deviation of the noise and mean value of the signal from these images, which is experimentally measured to be around 0.0106. As shown in Supplementary Fig. S5, we also characterize the optical system stability by measuring the speckle correlation, which remains greater than 0.995 over a duration of 30 minutes. After optimizing the setup, we use online Bayesian optimization to search for experimental hyperparameters with optical hardware in the loop. An example of the Bayesian optimization process during the experiment is illustrated in Supplementary Fig. S6.

Supplementary Note 3. Simulation comparison of optical NGRC and optical conventional RC based on scattering media

In the main text, we present the experimental results to show optical NGRC outperforms optical conventional RC based on scattering media in many aspects. Here we conduct additional numerical simulations to further compare these two architectures. We note that the best forecasting performance of the KS time series in simulation (without noise) based on optical conventional RC using scattering media is approximately 4 Lyapunov times, as reported in the Appendix of ref. [4]. By using optical NGRC in simulation, we achieve a forecasting capability up to 6 Lyapunov times with a significantly smaller reservoir and reduced training data. To quantitatively show their performance difference, we conduct more simulations as illustrated in Supplementary Fig. S7. At a specific reservoir size, we use the same training length (10,000 time steps) for both architectures to ensure a fair comparison between them, and repeat the simulation 25 times with different random matrices. Note that the warm-up period in the optical conventional RC is not considered into the training length, which is a bit unfair for the optical NGRC. We use the normalized root mean square error (NRMSE) over the first 2 Lyapunov times of the prediction as the metrics for comparison. As clearly shown in Supplementary Fig. S7, optical NGRC consistently achieves better performance (lower error) at all reservoir sizes ranging from 500 to 2,500, and also seems to show a smaller standard deviation than the optical conventional RC based on scattering media.

Supplementary Note 4. The impact of device quantization and noise on optical NGRC

Here we study the quantization effect of the SLM and camera devices on the optical NGRC performance. Due to the hardware constraint, quantization leads to data being represented with finite precision, thereby introducing errors into the encoding and detection processes throughout each iteration of the training and test phases. To quantify this effect, similar to Supplementary Note 3, we use the short-term prediction of the KS time series as the target task and calculate the NRMSE over the first 2 Lyapunov times in the prediction phase. With a reservoir size of 2,500 and a training length of 10,000 time steps, we examine the impact of quantization of two devices separately (by

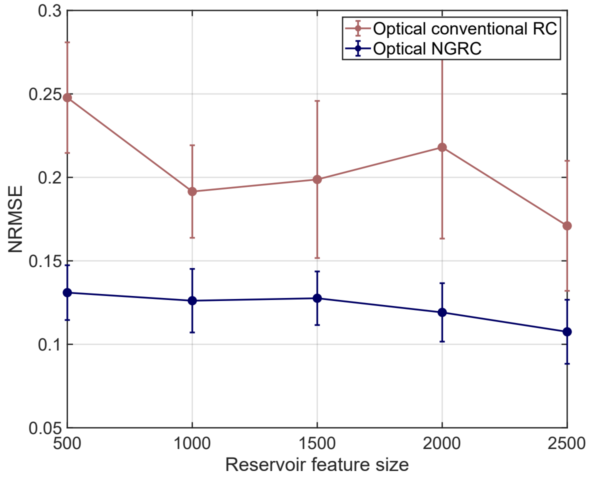

Figure S7: Simulated performance comparison of optical NGRC and optical conventional RC at different reservoir sizes. At each reservoir size, 25 different realizations are preformed with the same training length of 10,000 time steps. The error bar represents the range of one standard deviation.

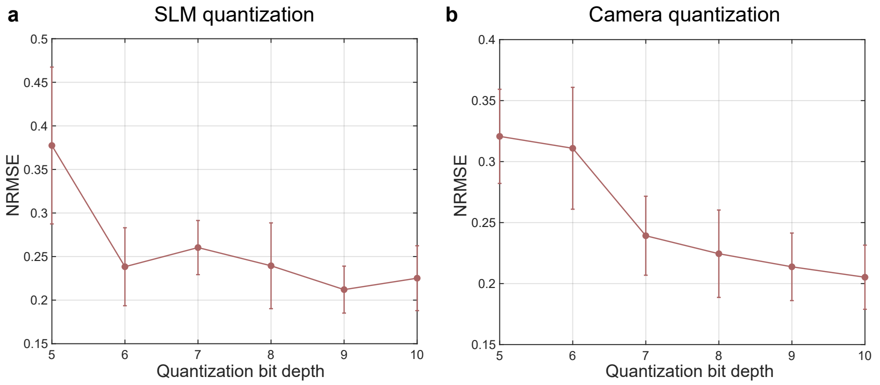

Figure S8: Simulation of optical NGRC forecasting errors based on different quantization bit depths of devices. a NRMSE versus quantization bit depth of SLM used for data encoding. **b** NRMSE versus quantization bit depth of camera used for reservoir feature measurement. At each quantization bit depth, 25 different realizations are preformed with a reservoir size of 2,500 and a training length of 10,000 time steps. The error bar represents the range of one standard deviation.

treating the other device free from quantization errors). As shown in Supplementary Fig. S8, it is expected that optical NGRC predicts better with lower errors with increased bit depths. As stated in the main text, our setup currently employs an effective bit depth of 7 bits for the SLM and 8 bits for the camera. This quantization level in the experiment is sufficient for the proof-of-concept

demonstrations (both short-term and long-term forecasting and NGRC observer) in this work.

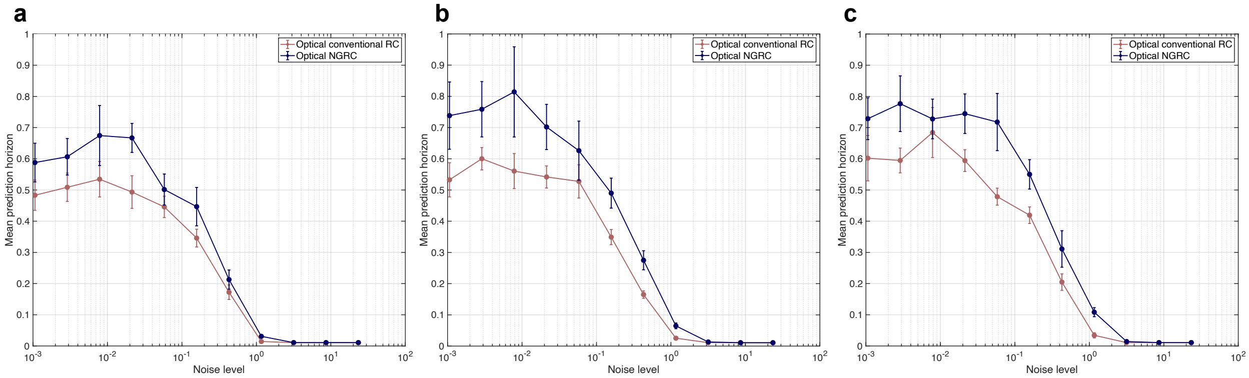

Figure S9: Simulated performance comparison of optical NGRC and optical conventional RC at different noise levels added to reservoir features. The noise level represents the ratio between the standard deviation of the added Gaussian noise and the mean value of the reservoir signal. The mean prediction horizon denotes the prediction length when NRMSE reaches a threshold of 0.2. The training length is 6,000, 12,000, and 18,000 time steps for **a**, **b**, and **c**, respectively.

The error bar indicates the range of one standard deviation from 40 repetitive realizations.

In addition, we also explore how the noise added to the reservoir features could impact the performance. Similar to Supplementary Fig. S7, here we compare the optical NGRC with conventional optical RC based on scattering media. As shown in Supplementary Fig. S9, we use the mean prediction horizon as the metric, characterized by the average prediction length when NRMSE reaches a threshold of 0.2. The reason why we use the mean prediction horizon here instead of NRMSE is that this metric is relatively easier to visualize, thanks to its bounded variance, which makes it more suitable for assessing the impact of noise. It can be seen that for both RC systems, an appropriate level of noise enhances forecasting performance due to noise regularization[14], but the prediction length decreases significantly when the noise is too strong. Also as shown in Supplementary Fig. S9, a longer training length is effective in mitigating the noise issue. Under almost all noise levels of interest, the optical NGRC outperforms (predicts longer than) conventional RC based on scattering media.

Supplementary Note 5. Optical computation analysis

Here we analyze the computational operations performed by the experimental setup and investigate its potential scaling properties. In this work, we exploit multiple light scattering to compute the reservoir features efficiently. As introduced in Supplementary Note 1, we perform a random projection optically described by a complex matrix $\boldsymbol{W} \in \mathbb{C}^{N\times M}$ where M and N are the number of input and output modes, respectively. We can break down each complex computational operation into its constituent real operations, wherein a complex multiplication is decomposed into 4 real multiplications and 2 real additions, and a complex addition entails 2 real additions[15]. We omit the element-wise computations related to encoding and detection nonlinearity since they correspond to a comparatively small number of operations. Given that the optical reservoir feature extraction encompasses NM complex multiplications and $N(M-1)$ complex additions, the equivalent total is $6NM + 2N(M-1) = 8NM - 2N$ real operations. For example, in the KS system forecasting experiments where $M = 64 \times 2 = 128$ (two time steps) and $N = 2,500$, the setup achieves approximately 0.1 giga floating point operations per second (GFLOPS) at a system frame rate of 40 Hz. Regarding the power consumption, in our experiment, the laser output power is approximately 2.5 mW, while the power usage is around 20 W for the SLM, 2.5 W for the camera, and 50 W for the control desktop computer, cumulating in a total power usage of 72.5 W. As a result, we estimate the computation energy efficiency as $\eta = 102,200,000/72.5025 \approx 1.41$ MegaOp J^{-1} (or equivalently 0.71 μJ Op^{-1}).

Improvements in energy efficiency can be achieved by reducing the power consumption of each component and/or increasing the overall frame rate of the system. This can be achieved by replacing the currently employed devices with more advanced alternatives. Currently we use only a small central region of the full screen of the SLM, therefore an SLM with less pixels but faster frame rate is preferred for our demonstration. For instance, using an one-dimensional SLM based on grating light valve can experimentally achieve 350 kHz modulation speed[16] and an electro-optic SLM can reach a GHz frame rate[17] and potentially increase the system's processing speed by orders of magnitude. Likewise, the system's overall frame rate also depends on the speed of the

camera, such that a faster detection device (e.g., a silicon photonic detector array with sub-GHz bandwidth[18] and a single-photon avalanche diode camera with sub hundred ps timing resolution[19]) could enhance the computational performance as well. In addition, the desktop computer used for managing the digital backend and communicating multiple devices can be replaced by a more energy-efficient electronic device, potentially with a power consumption below 10 W[20]. With these aforementioned hardware, we could envision a speed up of our system to at least kHz. Collectively, the total power consumption and the overall frame rate can be within approximately 10 (SLM from ref. [17]) $+10$ (FPGA from ref.[20]) $+2$ (Camera) $+0.0025$ (Laser) ≈ 22 W and reach 4 kHz (100 times higher than the current system), resulting in an energy efficiency of 2.2 nJ Op^{-1}. Note that the optical computation scale is determined by the pixel numbers of the SLM and camera used in the experiment. Luckily, the current technologies of SLMs and cameras support megapixels, which are already sufficient to show the optical computing advantage[4]. Moreover, even the aforementioned hardware optimization support million pixels, therefore the scaling can still be optimistically expected. Nevertheless, we cannot estimate the cost of the envisioned system as some of the devices are still under development in the laboratory. While the overall signal-to-noise ratio (SNR) of the system does not necessarily degrade when towards larger dimension and faster clock rate, the SNR of the system depends on the individual components used in the experiment. As a side note, we want to point out that replacing the SLM with a fast digital micromirror device (DMD) is not a viable solution here, as the encoding binarization could introduce large errors, thereby compromising performance particularly for challenging autonomous forecasting tasks. However, it is indeed interesting to explore the use of the fast and efficient system based on DMDs for other machine learning tasks rather than autonomous time-series forecasting, such as classification, based on optical NGRC in future studies.

Although we may not be able to compete with advanced commercial graphics processing unit in the current setting, such as NVIDIA V100 TENSOR CORE that achieves 0.27 TeraOp J^{-1} (3.7 pJ Op^{-1})[21], the optical NGRC features favourable scaling properties. In our system, the optical computation time and memory requirements almost do not scale with the reservoir dimension N, i.e. $O(1)$. In practice, they scale linearly with the reservoir size, i.e. $O(N)$, considering the digital-

to-analog and analog-to-digital conversions in the system. On the contrary, digital computers based on the von Neumann architecture exhibits quadratic scaling, i.e. $O(N^2)$, for matrix-vector multiplications. Therefore, we can foresee the optical setup will surpass digital computers in speed and efficiency beyond a certain data dimension threshold. This potential has been clearly evidenced by several previous experimental studies, where the benefits of optical computing emerge as N approaches the order of 10^4[4,7,22]. The current technologies of commercial SLMs and cameras are both at the megapixel (10^6 pixels) level. As such, we can encode a large amount of data in different sizes with a megapixel SLM and compute rich features with a megapixel camera. This can correspond to a $10^6 \times 10^6$ complex transmission matrix in our computing system, relating the input field at the SLM plane and output field at the camera plane. Computing the matrix vector multiplication at such a scale will be exhausted for digital computers in terms of both speed and memory costs.

The computational cost of a digital NGRC is quite different from optical NGRC. Firstly, considering an input data vector $\boldsymbol{u_t} \in \mathbb{R}^M$, if we build the NGRC feature vector $\boldsymbol{r_t}$ from K time steps and up to a polynomial order of H, the total number of feature terms is calculated as $N' = \sum_{i=0}^{H} \frac{(MK+i-1)!}{i!(MK-1)!}$. More precisely, for the low-dimensional Lorenz63 forecasting task in the main text, if we use $M = 3$, $K = 2$ and $H = 2$ as in ref. [12], the reservoir size will be $N' = 28$. Similarly, for predicting the KS time series, the reservoir size is calculated to be $N' = 8,385$ for $M = 64$, $K = 2$ and $H = 2$. As a result, the size of the reservoir feature N' increases polynomially with the data dimension M, and the ridge regression at a large reservoir size during training will become inefficient due to matrix inversion with complexity of $O(N'^3)$. And the total computational cost for each inference step can be estimated as $O(N'M)$. Heuristically speaking, the computational cost of digital NGRC increases polynomially when scaling to a large dimension since N' scales polynomially with M, if without prior knowledge of the data system to perform dimension reduction. A possible solution has been proposed to tackle this challenge, which employs a number of parallel NGRC to learn the large-scale KS time series[23]. In comparison, although the total computational complexity of optical NGRC is similarly $O(NM)$ dominated by the digital readout, N could be much smaller than that of the digital NGRC towards the large dimension. For instance, for the same KS prediction studied in this work, the digital NGRC would require the

reservoir size to be $N' = 8,385$, while we only need $N = 2,500$ in optical NGRC. Consequently, the scalability of optical NGRC in accommodating large input dimensions can be quite different from that of digital NGRC systems. As a side note, the polynomial feature terms in optical NGRC are generated naturally within each speckle feature on the camera, eliminating the need for the manual determination of the potential polynomial orders to be used. Instead, we simply train a linear readout layer to retrieve the most relevant feature terms. In optical NGRC, we can flexibly adapt the reservoir size depending on the difficulty of the task and the target performance. Yet, our optical NGRC approach requires a considerably larger reservoir size than the digital NGRC for small-scale datasets like the Lorenz63 system. This implies that we would need longer training length than the digital NGRC to obtain the readout matrix, as indicated in Supplementary Table S1. To tackle this issue, it will be beneficial to explore feature selection[24] on the experimentally generated reservoir feature in future studies.

Lastly, we discuss how the limitation of the feedback between the camera and the SLM impacts our current system. For the training phase, one advantage of our optical NGRC scheme compared to conventional optical RC[4] is that we do not require feedback between the camera and SLM. That is, we can obtain all the reservoir states in training without knowing previous reservoir states, thus alleviating the feedback bottleneck. In contrast, for conventional optical RC, one has to wait for $\boldsymbol{r}_t$ to be collected and processed, and then obtain $\boldsymbol{r}_{t+1}$ afterwards. This can be limited by the speed of communication between SLM and camera. For prediction (inference), such feedback bottleneck impacts differently for autonomous and non-autonomous prediction tasks. If we are dealing with autonomous forecasting tasks (Figs. 2 and 3 of the main text), we need to calculate the output $\boldsymbol{o}_{t+1}$ digitally with a trained readout matrix for each time step, based on the optically-generated reservoir state $\boldsymbol{r}_{t+1}$. And then we take this output as the input $\boldsymbol{u}_{t+1}$ for the next time step. As such, the feedback indeed impacts the autonomous forecasting tasks and slows down the overall frame rate of the system. But note that this digital and communication overhead scales linearly with the reservoir state sizes, as demonstrated in, e.g., Fig. 6 of ref.[4]. For large-scale reservoir size, it is still possible to achieve the optical advantage. If we are dealing non-autonomous tasks, for instance the observer task in Fig. 4 of the main text, where we do not have to wait for the previous

output to calculate the next output, we do not have this optical-electronic-optical feedback and are not constrained by such digital bottleneck just as the training phase, as opposed to optical conventional RC.

Supplementary Note 6. Comparisons between one-step prediction and autonomous prediction

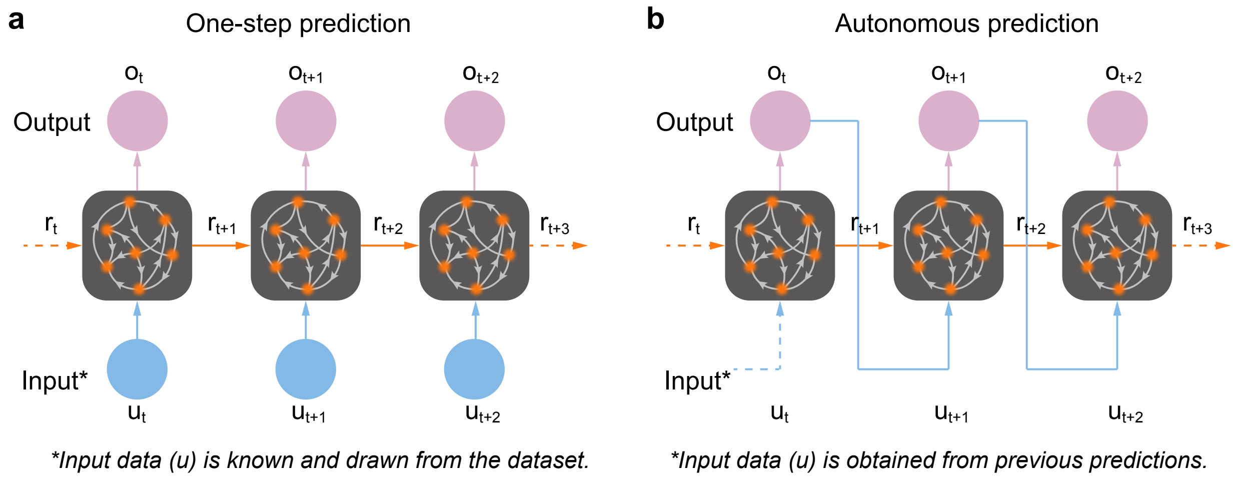

Figure S10: Illustration of differences between one-step prediction and autonomous prediction. a One-step prediction tasks, such as RC observers and one-step forecasting, rely on external input data at each time step. **b** Autonomous prediction tasks, such as short-term and long-term forecasting of a dynamical system, generate input data using the system's own previous predictions, forming a closed feedback loop.

Here we briefly introduce key concepts related to RC tasks in dynamical systems, which are essential for evaluating the model expressivity of different optical RC schemes. Broadly, though not exhaustively, there are two widely discussed categories as shown in Supplementary Fig. S10: one-step prediction and autonomous prediction. In one-step prediction tasks, during the inference stage, the reservoir input *at every time step* is directly drawn from the ground truth, and the trained readout layer maps the reservoir state to the desired target[25,26]. This task scheme uses accurate input data at every inference step, making it relatively easy to show good model expressivity of

physical RC systems. For instance, the optical NGRC observer results presented in Fig. 4 of this work, as well as main results of related works[27,28] (either inferring unmeasured state parameters or one next time step), fall into this category. In contrast, for autonomous prediction tasks illustrated in Supplementary Fig. S10b, during the inference stage, the input data at every time step is obtained from *the model's own previous prediction outputs*, therefore effectively closing a feedback loop. Since the previous prediction errors can impact the following predictions, this task scheme is notoriously challenging especially when dealing with chaotic time series forecasting. Therefore, it is often considered a more stringent test of an RC system's expressivity. For example, recent efforts in digital RC communities have successfully tackled tasks such as short-term prediction of large-scale spatiotemporal chaotic systems[23,29,30]. Our main results in Figs. 2-3 of the main text and Supplementary Figs. 7-9 focus on autonomous forecasting of chaotic dynamical systems, going well beyond the capabilities of state-of-the-art photonic RC systems. Such autonomous forecasting also serves as potential benchmarks for photonic RC systems, to fairly align with the standards in digital RC community.

In short, in addition to the inherent differences in optical computing mechanisms and scales between our work and refs. [27,28], we must emphasize that our work tackles more challenging RC tasks, i.e., autonomous prediction in both short-term and long-term, compared to one-step prediction in other works.

Supplementary Algorithms:

Algorithm 1: Optical NGRC for forecasting dynamical systems

Result: Predictions $\{\hat{\boldsymbol{o}}_t\} \in \mathbb{R}^{T_{test} \times M}$

Input: A training set $\{\boldsymbol{u}_t\} \in \mathbb{R}^{T_{train} \times M}$

Training: Prepare training ground truth $\{\boldsymbol{o}_t\} \in \mathbb{R}^{(T_{train}-2) \times M}$ based on $\boldsymbol{o}_t = \boldsymbol{u}_{t+2}$;

for $t = 2, 3, ..., T_{train}$ **do**

  Compute the SLM phase mask based on $[\boldsymbol{u}_t, \boldsymbol{u}_{t-1}, b]^T$;

  Run the optical experimental setup to retrieve the reservoir state $\boldsymbol{r}_{t+1} \in \mathbb{R}^N$;

end

Compute the output layer $\boldsymbol{W}_{out} \in \mathbb{R}^{M \times N}$ by minimizing

$\|\boldsymbol{W}_{out}\{\boldsymbol{r}_t\} - \{\boldsymbol{o}_t\}\|_2^2 + \beta\|\boldsymbol{W}_{out}\|_2^2$;

Prediction: Initialize a prediction starting point by specifying $\boldsymbol{u}_2$ and $\boldsymbol{u}_1$ as the last two time steps of the training set;

for $t = 2, 3, ..., T_{test} + 1$ **do**

  Compute the SLM phase mask based on $[\boldsymbol{u}_t, \boldsymbol{u}_{t-1}, b]^T$;

  Run the optical experimental setup to retrieve the reservoir state $\boldsymbol{r}_{t+1} \in \mathbb{R}^N$;

  Compute the prediction based on $\hat{\boldsymbol{o}}_{t+1} = \boldsymbol{W}_{out}\boldsymbol{r}_{t+1}$;

  Assign $\hat{\boldsymbol{o}}_{t+1}$ to $\boldsymbol{u}_{t+1}$;

end

Return the predictions $\{\hat{\boldsymbol{o}}_t\}$

Algorithm 2: Optical NGRC for deducing unmeasured variables of dynamical systems

Result: Predictions $\{\hat{\boldsymbol{o}}_t\} \in \mathbb{R}^{T_{test} \times Q}$

Input: A training input set $\{\boldsymbol{u}_t\} \in \mathbb{R}^{T_{train} \times P}$ with training ground truth $\{\boldsymbol{o}_t\} \in \mathbb{R}^{T_{train} \times Q}$, a test input set $\{\boldsymbol{v}_t\} \in \mathbb{R}^{T_{test} \times P}$

Training: Determine the number of input time steps as 5 spaced with a stride of 5;

for $t = 21, 22, ..., T_{train}$ **do**

  Compute the SLM phase mask based on $[\boldsymbol{u}_t, \boldsymbol{u}_{t-5}, \boldsymbol{u}_{t-10}, \boldsymbol{u}_{t-15}, \boldsymbol{u}_{t-20}, b]^T$;

  Run the optical experimental setup to retrieve the reservoir state $\boldsymbol{r}_t \in \mathbb{R}^N$;

end

Compute the output layer $\boldsymbol{W}_{out} \in \mathbb{R}^{Q \times N}$ by minimizing $\|\boldsymbol{W}_{out}\{\boldsymbol{r}_t\} - \{\boldsymbol{o}_t\}\|_2^2 + \beta\|\boldsymbol{W}_{out}\|_2^2$;

Prediction: Initialize the test starting point by drawing 5 time steps from the tail of the training input set as $[\boldsymbol{v}_{21}, \boldsymbol{v}_{16}, \boldsymbol{v}_{11}, \boldsymbol{v}_6, \boldsymbol{v}_1]$;

for $t = 21, 22, ..., T_{test} + 20$ **do**

  Compute the SLM phase mask based on $[\boldsymbol{v}_t, \boldsymbol{v}_{t-5}, \boldsymbol{v}_{t-10}, \boldsymbol{v}_{t-15}, \boldsymbol{v}_{t-20}, b]^T$;

  Run the optical experimental setup to retrieve the reservoir state $\boldsymbol{r}_t \in \mathbb{R}^N$;

end

Compute the prediction based on $\{\hat{\boldsymbol{o}}_t\} = \boldsymbol{W}_{out}\{\boldsymbol{r}_t\}$;

Return the predictions $\{\hat{\boldsymbol{o}}_t\}$

Supplementary Tables:

Supplementary Table 1: Comparison of optical NGRC, optical conventional RC based on scattering media and digital NGRC

Metrics	Digital NGRC[12]	Optical conventional RC[4]	Optical NGRC (this work)
Training length	Short[a]	Long (90,500 time steps for KS prediction)	Moderate (6,000 time steps for KS prediction)[b]
Warm-up before training	Very short (typically 2)	Long (typically $10^2 \sim 10^5$)	Very short (typically 2)
Number of hyperparameters	2[c]	6[d]	3[e]
Forecasting performance	Around 6 time units for Lorenz63[f]	Around 4 Lyapunov times for KS system	Around 2.5 Lyapunov times for KS sysem
Reservoir feature size	28 for Lorenz63 system	2,500 for KS system	10,000 for KS system
Model interpretability[g]	Interpretable	Uninterpretable	Interpretable
Physical openness[h]	Incompatible	Compatible	Compatible

[a] The training length of digital NGRC is shorter than conventional digital RC for low-dimensional chaotic time series processing[12,31], such as in Lorenz63 forecasting.

[b] The training length of optical NGRC is usually longer than that of digital NGRC due to the larger reservoir size and more readout parameters are used.

[c] The hyperparameters include the order of polynomials and ridge regularization parameter.

[d] The hyperparameters include the encoding scaling factors s_{in} and s_{res}, the encoding micropixel sizes p_{in} and p_{res}, the leaking rate, and the ridge regularization parameter[4].

[e] The hyperparameters include the relative weight η between two inputs, the bias b, and the ridge regularization parameter.

[f] The forecasting performance is comparable to the results previously achieved in ref.[31].

[g] It's important to note that the interpretability here is qualitative rather than quantitative. Specifically, it means that whether the reservoir computations can be understood by us. Interpreting conventional RC is challenging as highlighted by Jaeger in the book[32]. NGRC could be considered interpretable[12], as they exploit features from time-delayed inputs for applications.

[h] Physical openness refers to the compatibility and adaptability of the RC scheme to (other) physical hardware.

Supplementary Table 2: Summary of data encoding and processing parameters used in the experiments

**Parameters**	**Lorenz63 forecasting**[a]	**KS forecasting**[b]	**Lorenz63 observer**[c]	**KS observer**[d]
Input bias (b)	1.6	1.1	1.5	1.5
Relative weight (η)	7.5×10^{-1}	9.7×10^{-1}	1.0	1.0
Number of time steps	2	2	5	5
Encoding macropixel	28×28	7×7	28×28	21×21
Speckle grain size (pixels)	7	7	7	7
Reservoir size	2,000	2,500	2,000	2,500
Training length	4,000	6,000	4,00	10,000
Time interval (Δt)	0.025	0.25	0.025	0.25
Regularization parameter (β)	1.5×10^{-1}	5.6×10^{-1}	4.3×10^{-5}	3.4×10^{-1}

[a] The parameters used in short-term forecasting of Lorenz attractor in Fig. 2c.

[b] The parameters used in short-term forecasting of KS system in Fig. 3a.

[c] The parameters used in Lorenz63 observer in Fig. 4b.

[d] The parameters used in KS observer in Fig. 4c.

Table 3: Performance comparison with previous works on Lorenz63 and KS time-series prediction

**Reference**	**Lorenz63 forecasting**	**KS forecasting**
Gauthier et al. (Numerical)[12]	$\sim$ 6 time units	Not available
Pathak et al. (Numerical)[29]	Not available	$\sim$ 6 Lyapunov times
Pathak et al. (Numerical)[31]	$\sim$ 6 time units	$\sim$ 5 Lyapunov times
Vlachas et al. (Numerical)[30]	Not available	$\sim$ 4 Lyapunov times
Lu et al. (Numerical)[33]	$\sim$ 7 time units	Not available
Wikner et al. (Numerical)[34]	$\sim$ 4 time units	$\sim$ 2.5 Lyapunov times
Jiang et al. (Numerical)[35]	Not available	$\sim$ 6 Lyapunov times
Dong et al. (Numerical)[36]	Not available	$\sim$ 5 Lyapunov times
Antonik et al. (Experimental)[37]	$\sim$ 2 time units	Not available
Rafayelyan et al. (Experimental)[4]	Not available	$\sim$ 2.5 Lyapunov times
**This work (Experimental)**	$\sim$ 4.5 time units	$\sim$ 4 Lyapunov times

References

1. Gigan, S. Imaging and computing with disorder. *Nature Physics* **18**, 980–985 (2022).

2. Bingham, E. & Mannila, H. Random projection in dimensionality reduction: applications to image and text data. In *Proceedings of the seventh ACM SIGKDD international conference on Knowledge discovery and data mining*, 245–250 (2001).

3. Dong, J., Rafayelyan, M., Krzakala, F. & Gigan, S. Optical reservoir computing using multiple light scattering for chaotic systems prediction. *IEEE Journal of Selected Topics in Quantum Electronics* **26**, 1–12 (2019).

4. Rafayelyan, M., Dong, J., Tan, Y., Krzakala, F. & Gigan, S. Large-scale optical reservoir computing for spatiotemporal chaotic systems prediction. *Physical Review X* **10**, 041037 (2020).

5. Saade, A. *et al.* Random projections through multiple optical scattering: Approximating kernels at the speed of light. In *2016 IEEE International Conference on Acoustics, Speech and Signal Processing (ICASSP)*, 6215–6219 (IEEE, 2016).

6. Leonetti, M., Hörmann, E., Leuzzi, L., Parisi, G. & Ruocco, G. Optical computation of a spin glass dynamics with tunable complexity. *Proceedings of the National Academy of Sciences* **118**, e2015207118 (2021).

7. Pierangeli, D., Rafayelyan, M., Conti, C. & Gigan, S. Scalable spin-glass optical simulator. *Physical Review Applied* **15**, 034087 (2021).

8. Matthès, M. W., Del Hougne, P., De Rosny, J., Lerosey, G. & Popoff, S. M. Optical complex media as universal reconfigurable linear operators. *Optica* **6**, 465–472 (2019).

9. Launay, J. *et al.* Hardware beyond backpropagation: a photonic co-processor for direct feedback alignment. *arXiv:2012.06373* (2020).

10. Ghanem, H., Keriven, N. & Tremblay, N. Fast graph kernel with optical random features. In *ICASSP 2021-2021 IEEE International Conference on Acoustics, Speech and Signal Processing (ICASSP)*, 3575–3579 (IEEE, 2021).

11. Keriven, N., Garreau, D. & Poli, I. Newma: a new method for scalable model-free online change-point detection. *IEEE Transactions on Signal Processing* **68**, 3515–3528 (2020).

12. Gauthier, D. J., Bollt, E., Griffith, A. & Barbosa, W. A. Next generation reservoir computing. *Nature Communications* **12**, 5564 (2021).

13. Liang, X. *et al.* Physical reservoir computing with emerging electronics. *Nature Electronics* **7**, 193–206 (2024).

14. Estébanez, I., Fischer, I. & Soriano, M. C. Constructive role of noise for high-quality replication of chaotic attractor dynamics using a hardware-based reservoir computer. *Physical Review Applied* **12**, 034058 (2019).

15. Zhou, T. *et al.* Large-scale neuromorphic optoelectronic computing with a reconfigurable diffractive processing unit. *Nature Photonics* **15**, 367–373 (2021).

16. Tzang, O. *et al.* Wavefront shaping in complex media with a 350 khz modulator via a 1d-to-2d transform. *Nature Photonics* **13**, 788–793 (2019).

17. Trajtenberg-Mills, S. *et al.* Lnos: Lithium niobate on silicon spatial light modulator. *arXiv preprint arXiv:2402.14608* (2024).

18. Rogers, C. *et al.* A universal 3d imaging sensor on a silicon photonics platform. *Nature* **590**, 256–261 (2021).

19. Morimoto, K. *et al.* Megapixel time-gated spad image sensor for 2d and 3d imaging applications. *Optica* **7**, 346–354 (2020).

20. Li, Y., Li, S. E., Jia, X., Zeng, S. & Wang, Y. FPGA accelerated model predictive control for autonomous driving. *Journal of intelligent and connected vehicles* **5**, 63–71 (2022).

21. Khairy, M. Tpu vs gpu vs cerebras vs graphcore: a fair comparison between ml hardware (2020).

22. Ohana, R. *et al.* Kernel computations from large-scale random features obtained by optical processing units. In *ICASSP 2020-2020 IEEE International Conference on Acoustics, Speech and Signal Processing (ICASSP)*, 9294–9298 (IEEE, 2020).

23. Barbosa, W. A. & Gauthier, D. J. Learning spatiotemporal chaos using next-generation reservoir computing. *Chaos: An Interdisciplinary Journal of Nonlinear Science* **32** (2022).

24. Cai, J., Luo, J., Wang, S. & Yang, S. Feature selection in machine learning: A new perspective. *Neurocomputing* **300**, 70–79 (2018).

25. Lu, Z. *et al.* Reservoir observers: Model-free inference of unmeasured variables in chaotic systems. *Chaos: An Interdisciplinary Journal of Nonlinear Science* **27** (2017).

26. Yan, M. *et al.* Emerging opportunities and challenges for the future of reservoir computing. *Nature Communications* **15**, 2056 (2024).

27. Cox, N., Murray, J., Hart, J. & Redding, B. Photonic next-generation reservoir computer based on distributed feedback in optical fiber. *arXiv:2404.07116* (2024).

28. Wang, D., Nie, Y., Hu, G., Tsang, H. K. & Huang, C. A 103-tops/mm ˆ2 integrated photonic computing engine enabling next-generation reservoir computing. *arXiv preprint arXiv:2407.05840* (2024).

29. Pathak, J., Hunt, B., Girvan, M., Lu, Z. & Ott, E. Model-free prediction of large spatiotemporally chaotic systems from data: A reservoir computing approach. *Physical Review Letters* **120**, 024102 (2018).

30. Vlachas, P.-R. *et al.* Backpropagation algorithms and reservoir computing in recurrent neural networks for the forecasting of complex spatiotemporal dynamics. *Neural Networks* **126**, 191–217 (2020).

31. Pathak, J., Lu, Z., Hunt, B. R., Girvan, M. & Ott, E. Using machine learning to replicate chaotic attractors and calculate lyapunov exponents from data. *Chaos: An Interdisciplinary Journal of Nonlinear Science* **27** (2017).

32. Nakajima, K. & Fischer, I. *Reservoir Computing* (Springer, 2021).

33. Lu, Z., Hunt, B. R. & Ott, E. Attractor reconstruction by machine learning. *Chaos: An Interdisciplinary Journal of Nonlinear Science* **28** (2018).

34. Wikner, A. *et al.* Using data assimilation to train a hybrid forecast system that combines machine-learning and knowledge-based components. *Chaos: An Interdisciplinary Journal of Nonlinear Science* **31** (2021).

35. Jiang, J. & Lai, Y.-C. Model-free prediction of spatiotemporal dynamical systems with recurrent neural networks: Role of network spectral radius. *Physical review research* **1**, 033056 (2019).

36. Dong, J., Ohana, R., Rafayelyan, M. & Krzakala, F. Reservoir computing meets recurrent kernels and structured transforms. *Advances in Neural Information Processing Systems* **33**, 16785–16796 (2020).

37. Antonik, P., Haelterman, M. & Massar, S. Brain-inspired photonic signal processor for generating periodic patterns and emulating chaotic systems. *Phys. Rev. Appl.* **7**, 054014 (2017).
